# Physical exercise improved muscle strength and pain on neck and shoulder in military pilots

**DOI:** 10.3389/fphys.2022.973304

**Published:** 2022-09-02

**Authors:** Wei Heng, Feilong Wei, Zhisheng Liu, Xiaodong Yan, Kailong Zhu, Fan Yang, Mingrui Du, Chengpei Zhou, Jixian Qian

**Affiliations:** ^1^ Department of Orthopedics, Tangdu Hospital, Fourth Military Medical University, Xi’an, China; ^2^ 94333 Military Hospital, Shandong, China

**Keywords:** physical exercise, musculoskeletal disorders, neck pain, muscle strength, military pilots, meta-analysis

## Abstract

**Purpose:** To evaluate the effects of physical exercise on neck and shoulder muscle strength and pain in military pilots.

**Method:** Embase, PubMed, and Cochrane Library databases were searched studies published up to April 1, 2022. Studies that met the screening criteria were included in the final meta-analysis. We calculated neck and shoulder maximal voluntary isometric contractions (MVICs), prevalence of pain, and pain intensity. Heterogeneity was explored by subgroup and sensitivity analyses.

**Result:** A total of 15 studies with 907 participants were included. In the exercise group, muscle strength was significantly increased in four directions of neck motion: flexion (standardized mean difference (SMD) = 0.45; 95% CI, 0.08–0.82), extension (SMD = 0.63; 95% CI, 0.27–1.00), right lateral flexion (Rtflx) (SMD = 0.53; 95% CI, 0.12–0.94), and left lateral flexion (Ltflx) (SMD = 0.50; 95% CI, 0.09–0.91). Subgroup analysis showed that fighter pilots, strength plus endurance training, and a follow-up period <20 weeks exhibited more significant muscle strength improvements than helicopter pilots, simple strength training, and a follow-up period ≥20 weeks. Overall, the pooled odds ratio (OR) for the effect of physical exercise on the prevalence of neck pain was not statistically significant (I^2^ = 60%). Sensitivity analysis revealed that the heterogeneity was restored after removing each of two studies (I^2^ = 47%), and the pooled OR was statistically significant (OR = 0.46; 95% CI, 0.23 to 0.94, or OR = 0.47; 95% CI, 0.24–0.91). Furthermore, compared with observational studies (OS), the reduction in the prevalence of neck pain was more significant in randomized controlled trials (RCTs) (OR = 0.37; 95% CI, 0.18–0.78). No significant differences in the effects of exercise on shoulder muscle strength and neck and shoulder pain intensity were observed.

**Conclusion:** Physical exercise can improve neck muscle strength in military pilots. After removing studies that may be the source of heterogeneity, exercise showed a protective effect on neck pain, especially in RCTs. The conclusion that exercise had no effects on shoulder muscle strength and pain intensity should be taken with caution.

## 1 Introduction

Flight-related neck and shoulder pain is a common symptoms in military pilots (Espejo-Antunez et al., 2022). Neck disorders caused by flight have been afflicting pilots for a long time. Severe pain even leads to interruption or grounding of tasks, causing great harm to the physical and mental health of pilots (Nagai et al., 2014; Bahat et al., 2020).

Previous studies have shown that pain is affected by a variety of factors. In-flight effects, such as acceleration, sedentary behavior, head-worn equipment, and seatback, are all risk factors for neck pain and cervical spondylosis in pilots (Rintala et al., 2015; Verde et al., 2015; Posch et al., 2019). In particular, sudden movements of the pilot’s head upon exposure to high Gz accelerations increase the risk of acute cervical spine injury (Honkanen et al., 2018). Studies have shown that the neck muscles in pilots are significantly activated during flight, suggesting that the neck muscle is subjected to a high load (Sovelius et al., 2019). When the load is applied for a long period, the muscle becomes fatigued, which increases the risk of neck muscle strains (O'Conor et al., 2020). With the continuous improvement of aircraft performance, the load borne by pilots is also increasing. The acceleration of high-performance fighters can reach more than +9 Gz at present, which undoubtedly poses a greater challenge to pilot cervical spine health (Wallace et al., 2021).

Currently, measures to prevent flight-related neck and shoulder pain include warm-up and stretching before and after flight, head prepositioning, and exercise etc., all of which have been reported to provide protective effects (De Loose et al., 2008; Thoolen and van den Oord, 2015; O'Conor et al., 2020; Wallace et al., 2021). However, even so, the reporting rate of neck pain among pilots has remained high in recent years (Chumbley et al., 2017; Omholt et al., 2017; Posch et al., 2019). This increase rate is undoubtedly related to the improvement of aircraft performance. In addition, pain relief through special head positions in a confined cabin is not satisfactory (Rausch et al., 2021). The method of changing the design of seat backrest or cabin environment is not only time-consuming but also requires considerable economic investment. Under such a premise, the prevention and relief of neck and shoulder pain through one’s own exercise seems to be a relatively quick and effective method.

In many RCTs, strong evidence supports the effectiveness of physical exercise for neck pain (Sjøgaard et al., 2014; Saeterbakken et al., 2017; Park and Lee, 2020). The main purpose of exercise for pilots is to improve the ability to resist high Gz acceleration by improving the strength of the neck muscles and strengthening the control of the muscles (Rausch et al., 2021). In this study, we investigated the effects of physical exercise in military pilots on neck and shoulder muscle strength and pain base on a meta-analysis of previous studies, to provide more scientific and appropriate guidance for future training protocols.

## 2 Materials and methods

We followed the standards of PRISMA (Preferred Reporting Items for Systematic Reviews and Meta-Analyses) (Liberati et al., 2009; Zhang et al., 2021) and MOOSE (Meta-Analysis of Observational Studies in Epidemiology) (Stroup et al., 2000) guidelines during our research. The study protocol was registered with PROSPERO (International Prospective Register of Systematic Reviews: CRD42022336463).

### 2.1 Data sources and search strategy

The Embase, PubMed, and Cochrane Library databases were searched by three reviewers from inception to April 1st, 2022. Search items included medical subject headings (MeSH) and their following keywords: “pilot”, “exercise”, “training”, “neck”, and “shoulder”. The search strategy is provided in detail in Supplementary Table S1. Outcome measures were not used as search terms because reviewers wanted to comprehensively query relevant measures of exercising intervention in pilots to avoid omissions of important information. No language restrictions were employed during the search. Duplicate studies were removed by means of Endnote and manual secondary examination. During the whole process of literature retrieval, screening, assessment and data extraction, disagreements between the two reviewers were resolved by discussion with a third reviewer.

### 2.2 Selection criteria and data extraction

The literature screening process followed the PICOS principles, i.e., “population”, “intervention”, “comparison”, “outcome”, and “study”. The inclusion and exclusion criteria of the literature are provided in detail in Supplementary Table S2.

We extracted the following data from each included study: type of aircraft, total number of participants, age, height, weight, type of study, training site, training protocol, equipment, follow-up period, and outcomes. The primary outcomes were neck maximal voluntary isometric contractions (MVICs) (Lacio et al., 2021), including flexion, extension, left (Ltflx) and right (Rtflx) lateral flexion and the prevalence of neck pain (Ben Ayed et al., 2019). When included in our analysis, the units of the MVIC are unified and are typically reported as N or Nm. Given the paucity of studies, MVIC of shoulder elevation and pain intensity of neck and shoulder expressed by visual analog scale (VAS) (Kopsky et al., 2022) were used as secondary outcomes. The relative outcomes in the figures were analyzed with the aid of GetData Graph Digitizer 2.26.

### 2.3 Quality and risk-of-bias assessment

We used the Newcastle-Ottawa Quality Assessment Scale (NOS) to evaluate the risk of bias of the included observational studies (Stang, 2010). Of these, the NOS form has different terms for the case-control studies (CCS) and cohort studies. NOS scores ranged from 0 to 9, with a score of greater than 6 including a high-quality study. For randomized controlled trials (RCTs), we used the Cochrane Collaboration’s tool to assess the risk of bias in six domains: sequence generation, allocation concealment, blinding, incomplete outcome data, selective outcome reporting, and other issues (Page et al., 2018). Cross-sectional studies used an 11-item checklist with a full scale of 11 scores as recommended by the Agency for Healthcare Research and Quality (AHRQ) (Song et al., 2021) as follows: low quality = 0–3; moderate quality = 4–7; high quality = 8–11.

### 2.4 Data synthesis and statistical analysis

We used Review Manager (Revman) software (version 5.4) for quantitative analysis of the following variables between the exercise group (EG) and the control group (CG) or between the exposure group (EG) and nonexposure group (NEG): MVIC, prevalence of pain, and VAS. As a result of the unit difference (N or Nm), we calculated pooled estimates of the standard mean differences (SMDs) with 95% confidence intervals (CIs) for MVIC. Pooled outcomes of the same unit and VAS were calculated using the MD. We calculated pooled odds ratios (ORs) and 95% confidence intervals (CIs) for the prevalence of pain, which served as a categorical variable. Random-effects models were used for the analysis of all outcomes, and the I^2^ statistic was used to test for heterogeneity (Wei et al., 2022). Sensitivity analysis and subgroup analysis were performed on the results with I^2^ > 50% to identify the source of heterogeneity. Leave-one-out sensitivity analysis and subgroup analysis were performed to address heterogeneity as much as possible. Subgroups were classified according to the type of study, aircraft, equipment, training protocol, and follow-up period. *p* values <0.05 were considered statistically significant. Due to the small number of included studies in each outcome, we did not perform a test for publication bias (Sterne et al., 2011).

## 3 Results

### 3.1 Literature search and study selection

**FIGURE 1:**
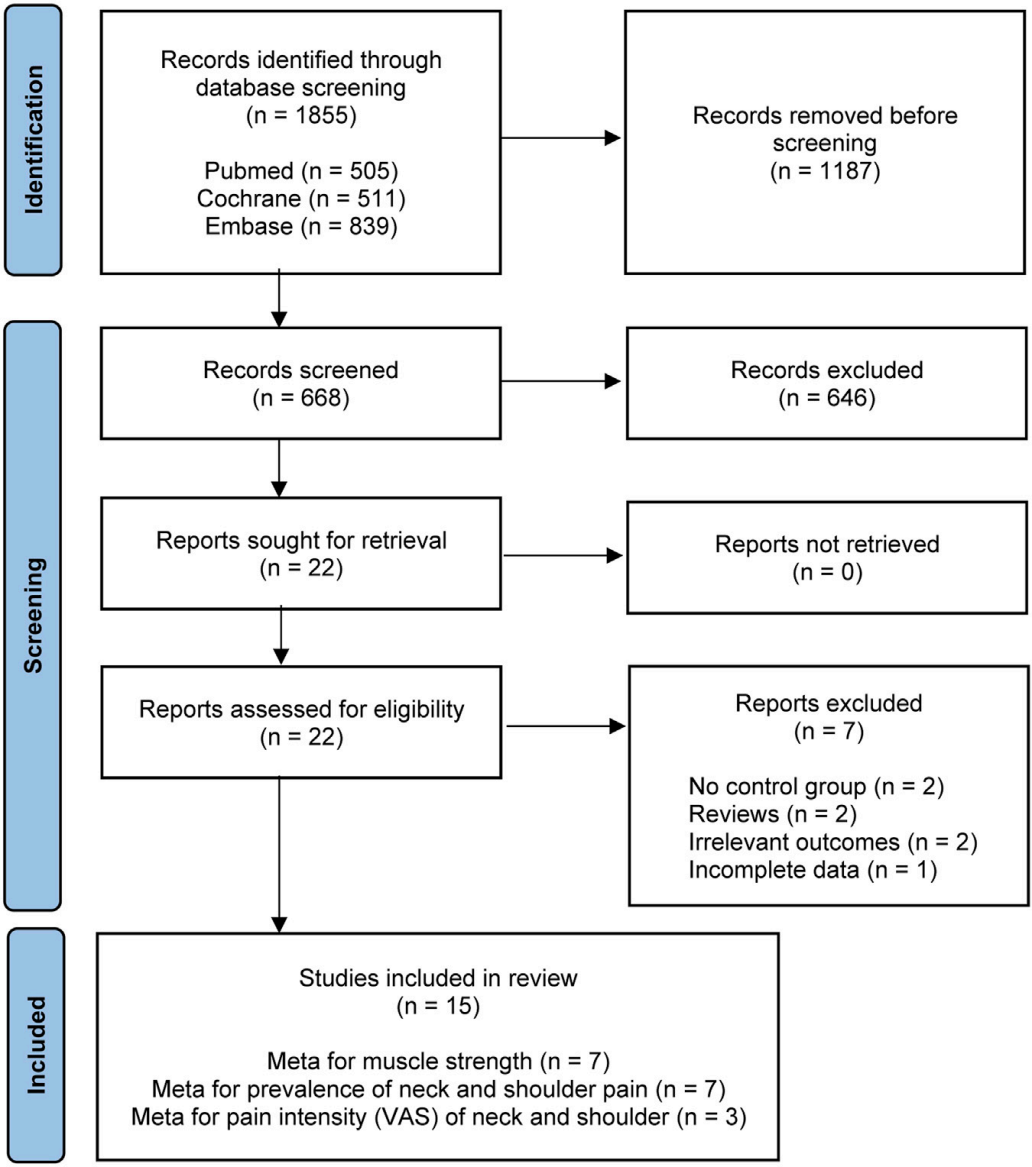
PRISMA flowchart of the study selection process for the meta-analysis.

Our search strategy identified a total of 1855 studies (PubMed = 505, Cochrane = 511, Embase = 839). After removing duplicates (*n* = 1186), 668 studies remained. After reading the titles and abstracts, 646 studies were excluded. Next, the full texts of 22 selected studies were reviewed. Seven studies were eliminated at this stage, and 15 studies were eligible for quantitative synthesis (Hämäläinen et al., 1993; Newman, 1997; Albano and Stanford, 1998; Jones et al., 2000; Alricsson et al., 2004; Burnett et al., 2005; De Loose et al., 2008; Ang et al., 2009; Lange et al., 2013; Salmon et al., 2013; Lange et al., 2014; Murray et al., 2017; Bahat et al., 2020; Murray et al., 2020; Rausch et al., 2021). The PRISMA flow chart for study selection is presented in Figure 1.

### 3.2 Quality assessment of the included studies

Supplementary Figure S1 shows a summary of the risk of bias assessment for RCTs (Alricsson et al., 2004; Burnett et al., 2005; Ang et al., 2009; Lange et al., 2013; Salmon et al., 2013; Lange et al., 2014; Murray et al., 2017; Bahat et al., 2020; Murray et al., 2020; Rausch et al., 2021). All studies were rated as high risk for the term “blinding of participants and personnel” due to the inability to conceal the intervention from the pilots who participated in the training program. We evaluated two case-control studies (Hämäläinen et al., 1993; De Loose et al., 2008) and two cohort studies (Newman, 1997; Albano and Stanford, 1998) using NOS. As a result, only one study was evaluated as high quality (total score = 6), two studies had a score of 5, and one study had a score of 4 (Supplementary Table S3). One cross-sectional study was evaluated using the 11-item checklist recommended by the AHRQ (Jones et al., 2000), and its quality was evaluated as moderate (total score = 4).

### 3.3 Study characteristics

The characteristics of the studies included in the meta-analysis are reported in Supplementary Table S4 **.** The study region included Denmark (*n* = 4) (Lange et al., 2013; Lange et al., 2014; Murray et al., 2017; Murray et al., 2020); United States (*n* = 2) (Albano and Stanford, 1998; Jones et al., 2000); Australia (*n* = 2) (Newman, 1997; Burnett et al., 2005); Sweden (*n* = 2) (Alricsson et al., 2004; Ang et al., 2009); Finland (*n* = 1) (Hämäläinen et al., 1993); Belgium (*n* = 1) (De Loose et al., 2008), Canada (*n* = 1) (Salmon et al., 2013); Israel (n = 1) (Bahat et al., 2020), and Germany (*n* = 1) (Rausch et al., 2021). A total of 907 pilots, including 675 (74.4%) fighter crew members and 232 (25.6%) helicopter pilots, were analyzed. The training protocol included muscle strength training alone (*n* = 5) (Albano and Stanford, 1998; Jones et al., 2000; De Loose et al., 2008; Bahat et al., 2020; Rausch et al., 2021), strength and endurance training (*n* = 5) (Hämäläinen et al., 1993; Alricsson et al., 2004; Burnett et al., 2005; Ang et al., 2009; Salmon et al., 2013), and a combination of strength, endurance, and coordination training (*n* = 5) (Lange et al., 2013; Salmon et al., 2013; Lange et al., 2014; Murray et al., 2017; Murray et al., 2020). The equipment used included hands-free devices, small devices (elastic rubber bands, dumbbells, or body blades), and complex devices (multi-cervical units (MCUs). Given that two training groups were included in each of two studies (Burnett et al., 2005; Salmon et al., 2013) using different training protocols or equipment, the studies were split into Group A and Group B for meta-analysis (Zhao et al., 2022).

### 3.4 Strength of neck muscles

**FIGURE 2:**
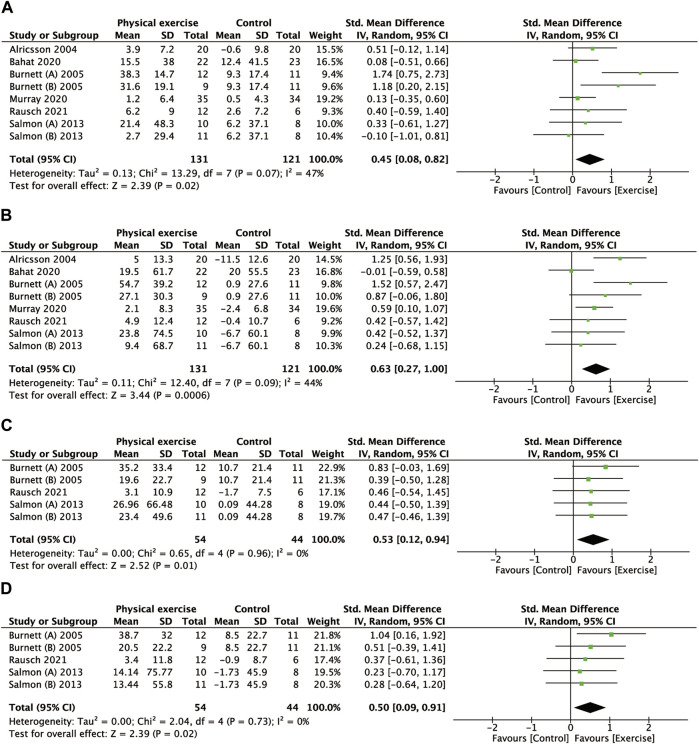
Forest plot comparing the MVIC of the neck in the physical exercise and control groups. **(A)** Flexion. **(B)** Extension. **(C)** Right lateral flexion. **(D)** Left lateral flexion. MVIC, maximal voluntary isometric contractions.

Figure 2 shows the forest plot of the relationship between physical exercise and muscle strength. The results showed that the increase in muscle strength was more significant in the exercise group in the four directions of neck movement: flexion (SMD, 0.45; 95% CI, 0.08 to 0.82; I^2^ = 47%) (Figure 2A), extension (SMD, 0.63; 95% CI, 0.27 to 1.00; I^2^ = 44%) (Figure 2B), Rtflx (SMD, 0.53; 95% CI, 0.12 to 0.94; I2 = 0%) (Figure 2C), and Ltflx (SMD, 0.50; 95% CI, 0.09 to 0.91; I^2^ = 0%) (Figure 2D). The results of the unified unit of the muscle strength showed a higher change in the exercise group than the control group: 17.53 N (95% CI, 5.68 to 29.39; I^2^ = 35%) in flexion (Supplementary Figure S2A); 27.55 N (95% CI, 7.11 to 47.99; I^2^ = 35%) and 8.54 Nm (95% CI, 0.57 to 16.50; I^2^ = 72%) in extension (Supplementary Figure S3); 17.08 N (95% CI, 3.59 to 30.56; I^2^ = 0%) in Rtfix (Supplementary Figure S4); 19.25 N (95% CI, 5.54 to 32.96; I^2^ = 0%) in Ltflx (Supplementary Figure S5), except for the flexion in Nm (MD, 1.60; 95% CI, -0.62, 3.81; I^2^ = 0%) (Supplementary Figure S2B).

The subgroup analysis showed that muscle strength increases in neck flexion (SMD, 1.06; 95% CI, 0.32 to 1.80; I^2^ = 56%) (Supplementary Figure S6), neck extension (SMD, 1.22; 95% CI, 0.74 to 1.69; I^2^ = 0%) (Supplementary Figure S10), and Ltflx (SMD, 0.78; 95% CI, 0.15 to 1.41; I^2^ = 0%) (Supplementary Figure S17) were significantly greater than those noted in the control group, and Rtflx (SMD, 0.62; 95% CI, 0.00 to 1.24; I^2^ = 0%) (Supplementary Figure S14) approached a significant higher value. In contrast, the helicopter group exhibited no significant difference except for extension (SMD, 0.49; 95% CI, 0.12 to 0.85; I^2^ = 0%) (Supplementary Figure S10). In addition, except for the significant heterogeneity of the aircraft type in neck extension (*p* = 0.004) (Supplementary Figure S10), no significant heterogeneity in other directions was noted.

The subgroup analysis of training equipment showed that the strength increase in neck flexion (SMD, 0.33; 95% CI, 0.33 to 0.63; I^2^ = 0%) and extension (SMD, 0.68; 95% CI, 0.37 to 0.98; I^2^ = 0%) in the exercise group using a small device was significantly greater than that in the control group (Supplementary Figures S7, S11). However, the strength increase in Ltflx and Rtflx was not significantly different (Supplementary Figures S15, S18). In addition, the strength increase using the complex device was also significant in the neck flexion (SMD, 1.74; 95% CI, 0.75–2.73) (Supplementary Figure S7), extension (SMD, 1.52; 95% CI, 0.47–2.47) (Supplementary Figure S11), and Ltflx (SMD, 1.04; 95% CI, 0.16–1.92) (Supplementary Figure S18) with near significance in Rtflx (SMD, 0.83; 95% CI, -0.03–1.69) (Supplementary Figure S15). Training equipment had significant heterogeneity in neck flexion (*p* = 0.01) and extension (*p* = 0.02) (Supplementary Figures S7, S11).

The subgroup analysis of the training protocol showed that muscle strength in the strength plus endurance training group was significantly greater than simply strength training and comprehensive (strength+endurance+coordination) training in neck flexion (SMD, 0.80; 95% CI, 0.07 to 1.52; I^2^ = 65%) (Supplementary Figure S8), extension (SMD, 0.70; 95% CI, 0.47 to 1.51; I^2^ = 32%) (Supplementary Figure S12), Rtflx (SMD, 0.57; 95% CI, 0.06 to 1.09; I^2^ = 0%) (Supplementary Figure S16), and Ltflx (SMD, 0.62; 95% CI, 0.10 to 1.14; I^2^ = 0%) (Supplementary Figure S19). The comprehensive training protocol was significant only in extension strength (SMD, 0.55; 95% CI, 0.12 to 0.98; I^2^ = 0%) (Supplementary Figure S12), and the other protocols were not significantly different. The heterogeneity of the training protocol was not statistically significant.

The subgroup analysis of the follow-up period showed that there were statistically significant muscle strength increases in neck flexion (SMD, 0.55; 95% CI, 0.01 to 1.10; I^2^ = 59%) (Supplementary Figure S9) and neck extension (SMD, 0.52; 95% CI, 0.06 to 0.97; I^2^ = 39%) (Supplementary Figure S13) at a follow-up period of less than 20 weeks. Only the strength increase in extension (SMD, 0.87; 95% CI, 0.23 to 1.51; I^2^ = 59%) at a follow-up period of no less than 20 weeks was statistically significant (Supplementary Figure S13). The heterogeneity of the follow-up period was not statistically significant.

### 3.5 Prevalence of neck pain

**FIGURE 3:**
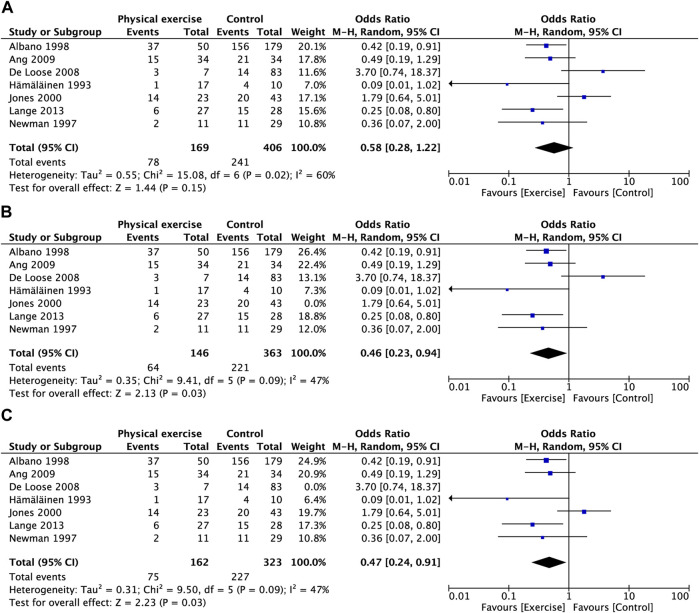
Forest plot comparing the prevalence of neck pain in the physical exercise and control groups. **(A)** Before leave-one-out sensitivity analysis **(B)** After leave-one-out sensitivity analysis (Jones et al., 2000). **(C)** After leave-one-out sensitivity analysis (De Loose et al., 2008).

**FIGURE 4:**
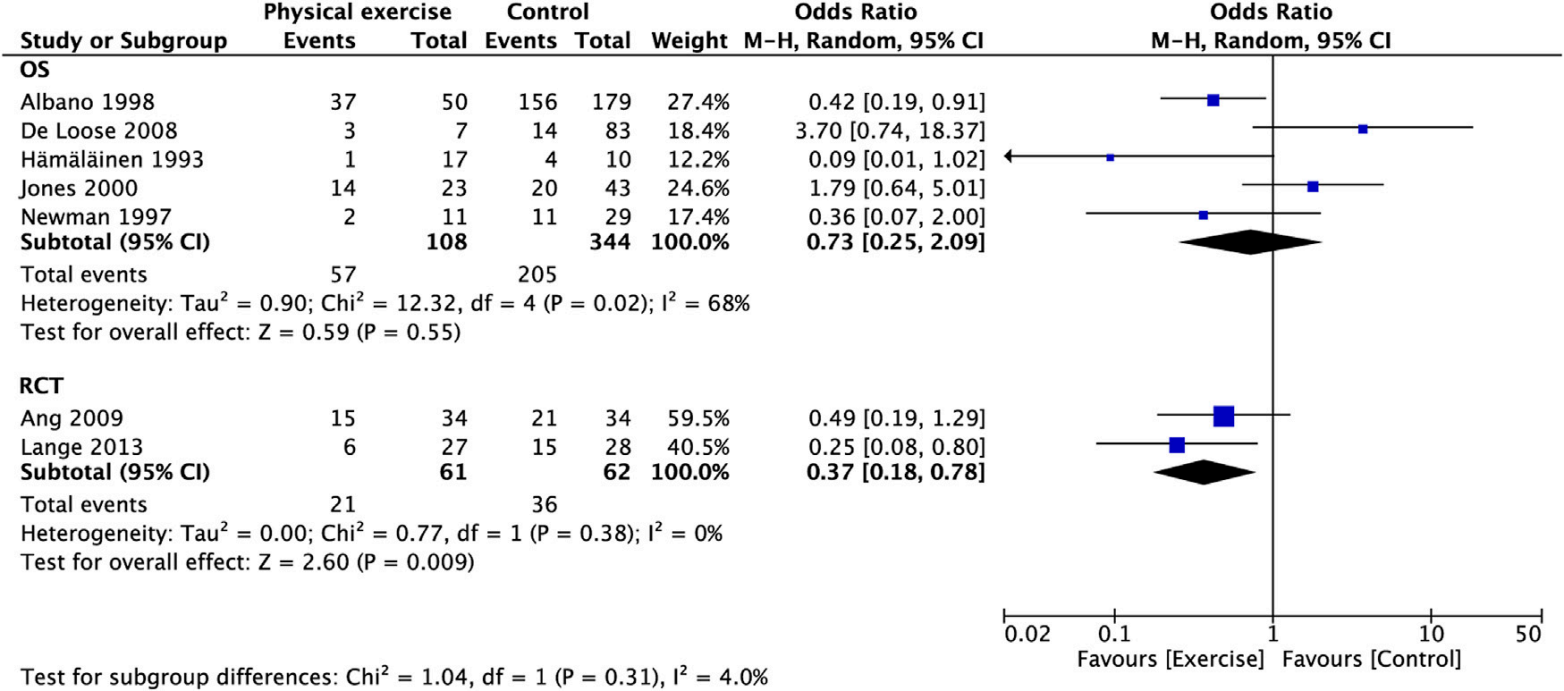
Subgroup analysis for prevalence of neck pain (type of study).

**FIGURE 5:**
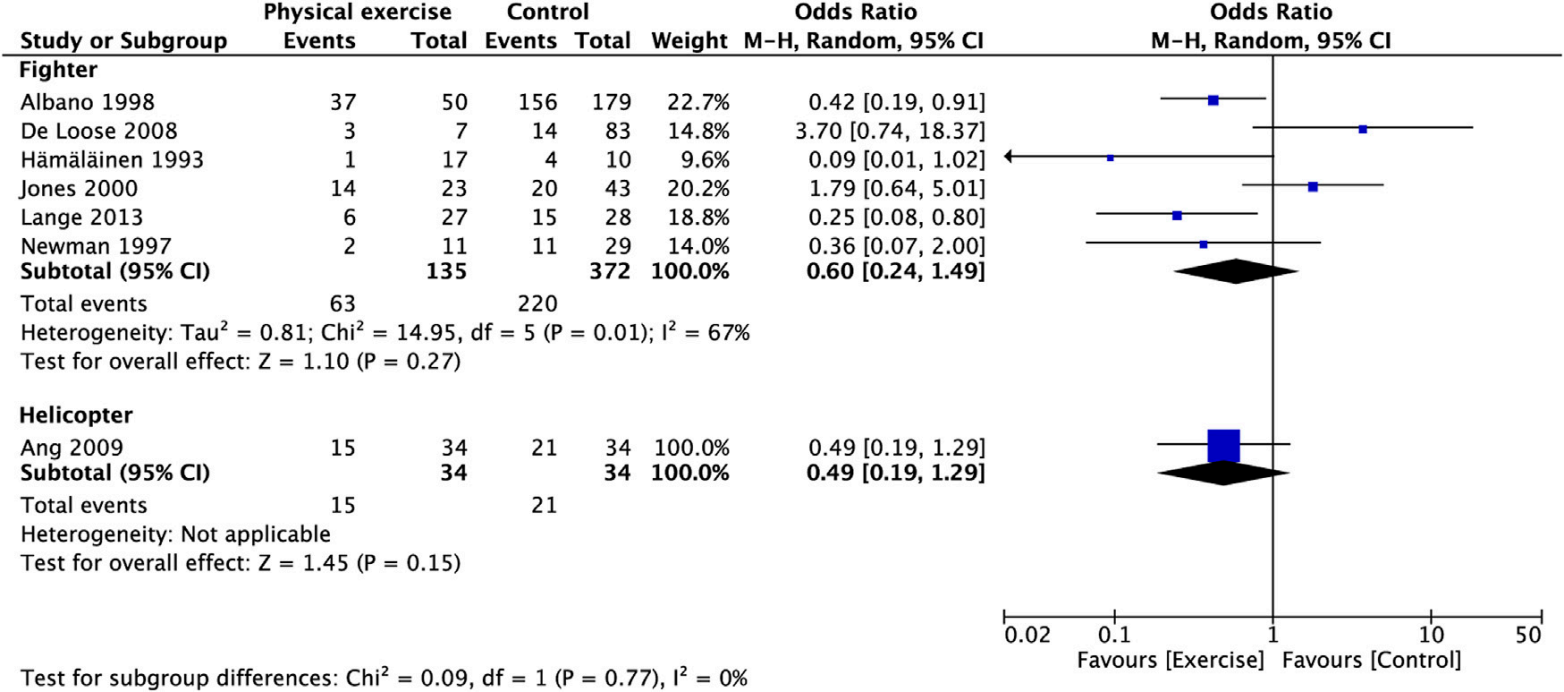
Subgroup analysis for prevalence of neck pain (type of aircraft).

**FIGURE 6:**
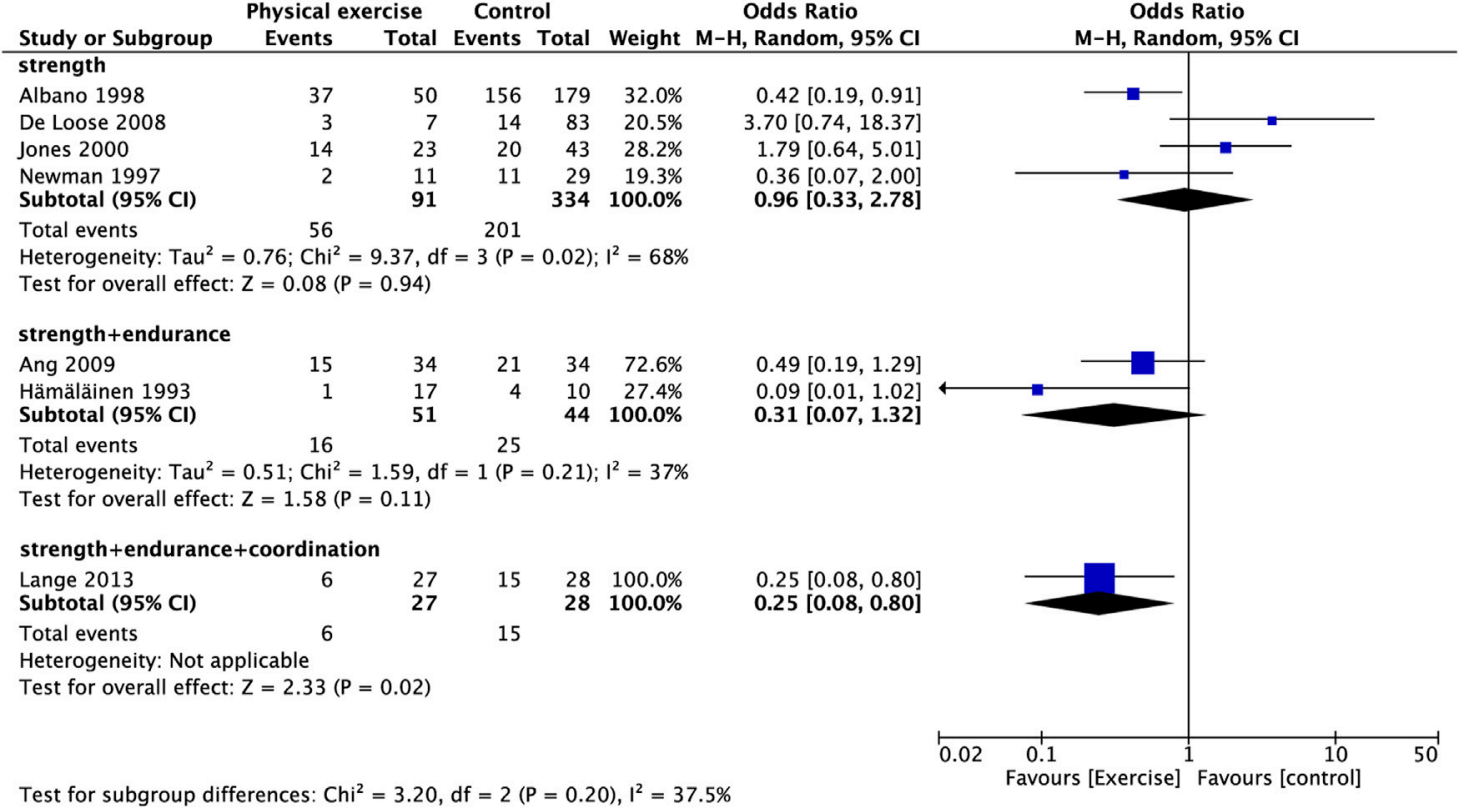
Subgroup analysis for prevalence of neck pain (training protocol).

Figure 3A presents the relationship between physical exercise and the prevalence of neck pain. The results showed that the prevalence of neck pain in the exercise group was not statistically significant compared with that in the control group (OR, 0.58; 95% CI, 0.28 to 1.22; I^2^ = 60%). Sensitivity analysis showed that heterogeneity was restored (I^2^ = 47%) after the exclusion of two studies (Jones et al., 2000; De Loose et al., 2008), and the pooled OR showed a significant association between physical exercise and the prevalence of neck pain (OR, 0.46; 95% CI, 0.23–0.94) (Figure 3B) (OR, 0.47; 95% CI, 0.24–0.91) (Figure 3C). The subgroup analysis of study type showed that the reduction in the prevalence of neck pain with the RCT compared with OS was statistically significant (OR, 0.37; 95% CI, 0.18 to 0.78; I^2^ = 0%) (Figure 4). The prevalence of neck pain was not significant based on the type of aircraft (Figure 5). In the training protocol subgroup, although comprehensive training (strength + endurance + coordination) was significant (OR, 0.25; 95% CI, 0.08–0.80) (Figure 6), the result was relatively conservative due to the small number of studies (n = 1). In addition, the prevalence of neck pain was not significant for the heterogeneity of all subgroups.

### 3.6 Strength of shoulder muscles

**FIGURE 7:**
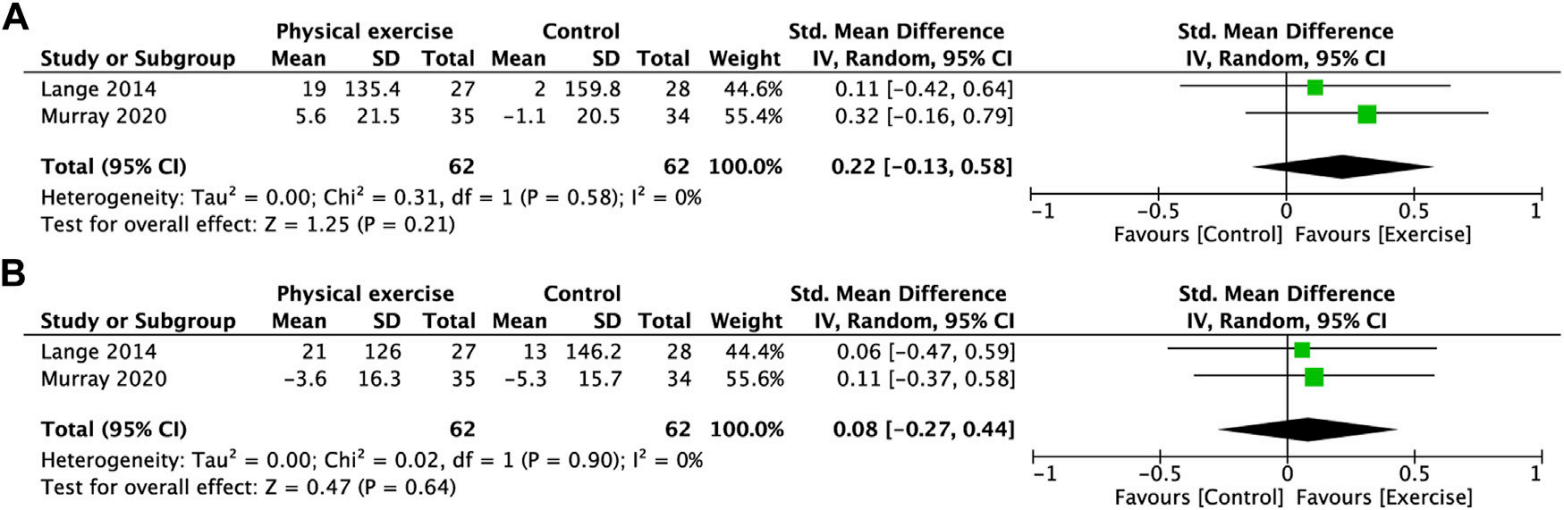
Forest plot comparing MVIC of the shoulder in the physical exercise and control groups. **(A)** Right elevation. **(B)** Left elevation. MVIC, maximal voluntary isometric contractions.

Figure 7 shows that physical exercise did not significantly improve right shoulder (Figure 7A) or left shoulder muscle strength (Figure 7B) compared with the control group.

### 3.7 Pain intensity of the neck and shoulder

**FIGURE 8:**
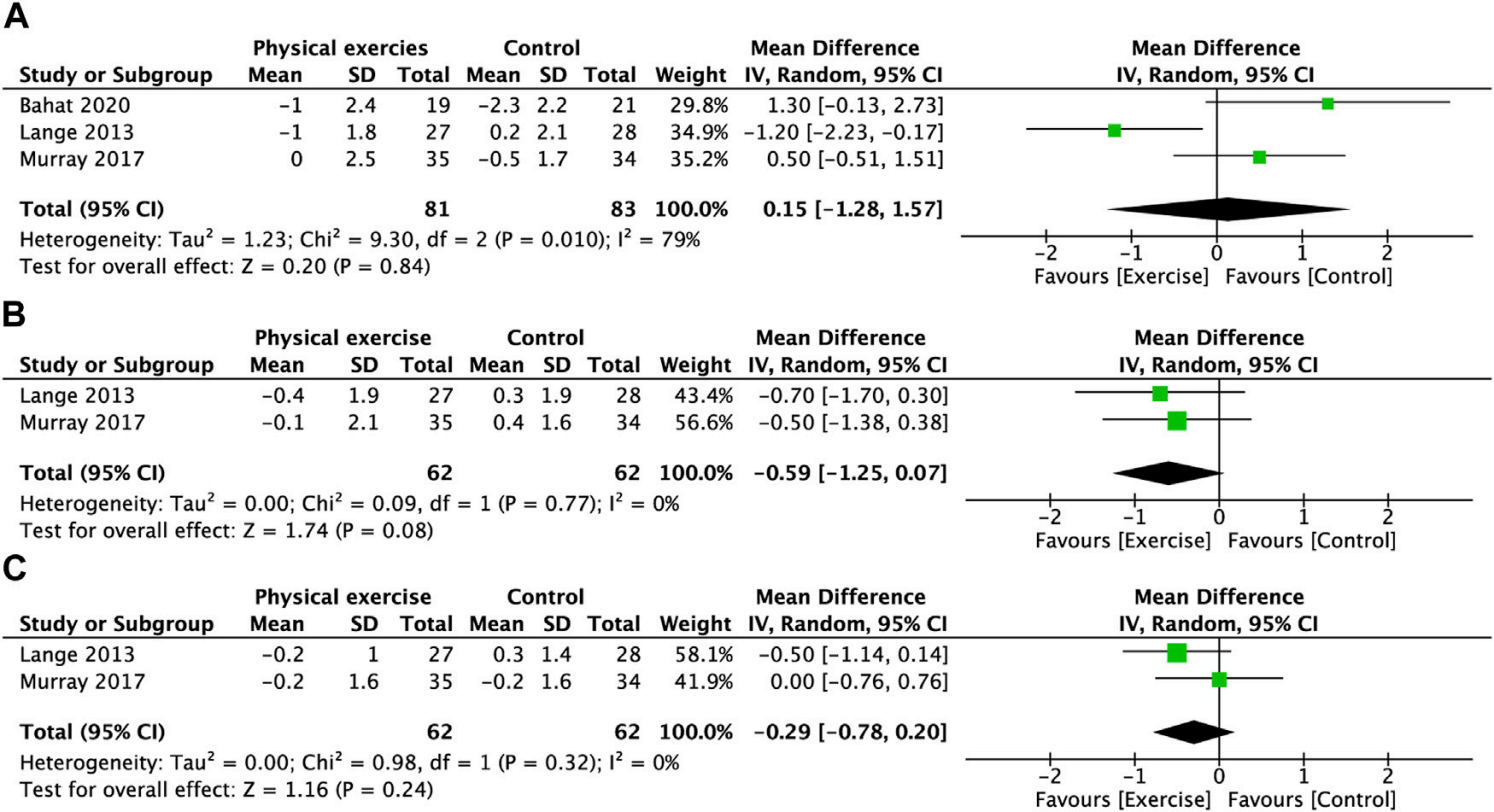
Forest plot comparing the VAS scores of the neck and shoulder in the physical exercise and control groups. **(A)** Neck. **(B)** Right shoulder. **(C)** Left shoulder. VAS, visual analog scale.

Figure 8 shows that physical exercise did not significantly reduce pain intensity in the neck (Figure 8A), right shoulder (Figure 8B), or left shoulder (Figure 8C) compared with the control group.

## 4 Discussion

### 4.1 Association between exercise and neck and shoulder strength in pilots

Methods to improve neck muscle strength through exercise have become a consensus in the general working population. However, due to the particularity of a pilot’s profession, daily flights and routine training occupy most of his/her day. Can additional training programs significantly improve neck and shoulder muscle strength, and does training have the same effect on muscle strength in different parts of the neck? These issues need to be clarified urgently because they will help us to further develop more scientific training programs. Currently, there is no evidence-based exercise program that can specifically improve the strength of muscles around the spine to reduce physiological and perceived stress during high Gz flight (Rausch et al., 2021). Fortunately, our meta-analysis yielded positive results showed that physical exercise significantly improved MVIC in the four directions of neck motion in flexion, extension, Rtflx, and Ltflx (Figure 2), indicating that exercise had a significant effect on neck muscle strengthening in pilots. In particular, the pooled estimates of the MD in unified unit showed that the muscle strength change in neck flexion increased by 17.53 N in the exercise group compared to the non-exercise group; the neck extension increased by 27.55 N, and the exercise group could do more work by 8.54 Nm with the same torque; the muscle strength in right and left flexion increased by 17.08 N and 19.25 N respectively. Although no significant result was obtained for muscle strength change in neck flexion in Nm, in general, exercise did increase the pilots’ neck muscle strength to some extent (Supplementary Figure S2-5).

However, the difference in shoulder elevation was not significant (Figure 7). Murray et al. (Murray et al., 2020) found that the shoulder musculature was stronger in pilots and crew than in general working staff, whereas neck strength was not correspondingly higher. Our results also seem to corroborate his study in that the benefits obtained by neck muscles through exercise may be more pronounced than those of the shoulders. It has been demonstrated that the trapezius muscle is activated during air combat maneuvers, especially when fighter pilots adopt a specific head position (Netto and Burnett, 2006). This finding also suggests that our training protocol needs to strengthen the intensity on the shoulder to obtain significant muscle strength improvement. However, because the number of studies was too small, we remain cautious about this conclusion.

### 4.2 Association between exercise and neck and shoulder pain in pilots

Although many studies have demonstrated that physical exercise can relieve work-related neck pain (Blangsted et al., 2008; Sjøgaard et al., 2014; Saeterbakken et al., 2017; Chen et al., 2018; Park and Lee, 2020), there is no strong evidence that physical exercise reduces the prevalence or intensity of flight-related neck and shoulder pain because the different mechanisms contrast with the occupational pain in general high Gz and special aviation environments are the root causes. It is worth affirming that there are still many OS and RCTs with positive conclusions. Albano et al. (Albano and Stanford, 1998) investigated 268 American F-16 pilots and found that the probability of neck injury was reduced in the early exercise intervention (*p* = 0.024). Ang et al. (Ang et al., 2009) performed 1-year neck training in 68 helicopter pilots and found that the incidence of neck pain was reduced in the past week (OR, 3.2; 95% CI, 1.3–7.8) and the past 3 months (OR, 1.9; 95% CI, 1.2–3.2) after follow-up. Lange et al. (Lange et al., 2013) also showed that targeted neck exercises for fighter pilots can reduce their neck pain intensity. However, there are also studies that report the opposite conclusions. De Loosede et al. (De Loose et al., 2008), Newman et al. (Newman, 1997), and Jones et al. (Jones et al., 2000) showed no difference in the incidence of neck pain between the exercise and no exercise groups. In-flight acceleration loading increases the pull to the spine through the muscles and ligaments of the neck, accelerating disc degeneration and the development of neck pain (Iatridis et al., 2013). In contrast, pilots with neck pain had significantly lower muscle strength (Ang et al., 2005). More studies have shown that increasing the strength of neck muscles can yield more sustained muscle support and protect the spine from high Gz-induced injury (Green and Brown, 2004; Stergiou et al., 2017; Sovelius et al., 2019). Second, a stronger muscle reduces the transmission of force, enhances the stability of the spine, and reduces the risk of load-induced premature disc degeneration (Harrison et al., 2015; Sovelius et al., 2020).

Our pooled OR showed no significant effect of physical exercise in reducing the prevalence of neck pain (Figure 3A). However, we noted high heterogeneity in this result (I^2^ = 63%). Fortunately, we found possible sources of heterogeneity through sensitivity analysis. After excluding the studies by Jones et al. (Jones et al., 2000) and De Loose et al. (De Loose et al., 2008), heterogeneity was restored (I^2^ = 47%), and the pooled OR showed a significant association between physical exercise and the prevalence of neck pain (Figures 3B,C). The study by Jones et al. (Jones et al., 2000) was a cross-sectional study and was lower in level of evidence than RCTs, cohort studies, and case-control studies according to Oxford Centre for Evidence-Based Medicine (Howick and Glasziou, 2011). De Loose et al. (De Loose et al., 2008) had a smaller sample size, especially in the exposure group. Only seven people participated in exercise. Although only three people in the exposure group had neck pain, which may seem like a small number, the smaller denominator resulted in a seemingly increased prevalence. In addition, the study was a case-control study with no a high-level of evidence (Howick and Glasziou, 2011). Our subgroup analysis of study types revealed that RCTs had a significantly lower prevalence of neck pain, but not for the OS (Figure 4). Given that OS are retrospective, the exercises were unsupervised, and a large recall bias was noted in the respondents. Second, we noted a large difference in reporting pain rates at baseline across studies, which was attributed to the different definitions of neck pain, which also contributed to heterogeneity to some extent.

Our analysis for pain intensity showed no difference in pain scores between the exercise and control groups, either in the neck or shoulder (Figure 8). However, the findings are cautious due to the small number of studies. The investigation by Lange et al. (Lange et al., 2013) showed that although a significant majority (82%) of pilots experienced neck pain in the year, none experienced symptoms on a daily basis. Seventy-three percent of the pilots had a pain frequency of <31 days in a year. Bahat et al. (Bahat et al., 2020) also expressed a consistent view that flight-related neck pain usually occurs acutely after a flight and recovers over several days. This also suggests that pilot neck pain is not chronic, but periodically occurs with exposure. This cyclical pain would result in lower mean scores because the odds of experiencing pain in such a short duration are low. Murray et al. (Murray et al., 2016) suggested that the “dilution” effect of participants without pain results in lower pain scores at baseline and a lower likelihood of reducing pain intensity through exercise. Some studies have used the percentage of individuals with a 50% reduction in VAS (Salmon et al., 2013) as an outcome indicator to evaluate the effect of exercise, which seems to be an option. In addition, we believe that the reporting of pain frequency may be more representative of the characteristics of cyclical pain than intensity and prevalence.

### 4.3 Association between subgroups and outcome

#### 4.3.1 Type of aircraft

Our subgroup analysis found that the muscle strength changes in fighter pilots after exercise were more significant than those of helicopter pilots (Supplementary Figures S6, S10, S14, S17). Studies on the differences in muscle strength of pilots of different aircraft types are limited. Ang et al. (Ang et al., 2005) measured the MVC of the neck muscle in pilots with neck pain and found that muscle strength was decreased in fighter pilots with neck pain, but not in helicopter pilots. The source of the difference may be that fighter pilots are frequently exposed to high Gz loads, resulting in decreased skeletal muscle stress. Helicopter pilots have a long driving time, and long fixed postures more often manifest as muscle fatigue. Fighter pilots may have generally lower neck strength than helicopter pilots, which may explain our results. Specially, fighter pilots may have more room to improve their neck strength due to reduced muscle strength caused by a high prevalence of neck pain. Because pilots in different types of aircraft have different types of exposure and pain, the training will also have a different focus (Ang et al., 2005; Murray et al., 2015). Helicopter pilots are more suitable for endurance exercise due to frequent neck muscle fatigue.

The prevalence of neck pain varies among different types of aircraft. Grossman et al. (Grossman et al., 2012) found that neck pain is more common in fighter pilots compared with attack helicopter and transport aircraft pilots possibly because the necks of fighter pilots are more susceptible to a combination of factors, such as helmets, acceleration, and sitting posture. De Loose et al. (De Loose et al., 2008) stated that “check six” is a routine flight maneuver for fighter pilots and the most common cause of neck pain in investigated pilots. This action requires the combination of lateral neck flexion and extension muscles to achieve maximal rotation of the neck. Therefore, the neck muscles of fighter pilots are affected by greater flight stress than other types of pilots. In summary, the incidence of neck pain was greater in fighter pilots compared with helicopter pilots. We included seven studies in our analysis on the prevalence of neck pain, six of which focused on fighter pilots and only one study focused on helicopter pilots, which resulted in a high baseline in the prevalence of neck pain. Due to the paucity of studies on helicopter pilots, we did not observe a difference in the effect of exercise on the prevalence of neck pain in pilots of different aircraft types (Figure 5).

### 4.3.2 Equipment

A commonly used device for neck training is an elastic band named the Thera-band (THER) (Burnett et al., 2005; Ang et al., 2009) (2009, Ang)(Salmon et al., 2013; Murray et al., 2020), which is also referred to as a headband (Lange et al., 2013) or rubber tube in some literature (Alricsson et al., 2004). In general, one end of the elastic band is fixed to a helmet or a harness over the head, and the other end is fixed or attached to a weight. The trainer then performs muscle contractions in different directions of neck movement. This equipment is also suitable for shoulder training (Murray et al., 2020) or is replaced with other weights, such as dumbbells (Lange et al., 2013). In most studies, muscle strength increased and pain improved in pilots who applied elastic bands (Alricsson et al., 2004; Lange et al., 2013; Salmon et al., 2013; Murray et al., 2020), but some studies also yielded negative results (2017, Murray). Our subgroup analysis is consistent with most studies, where the muscle strength increase in neck flexion and extension with small devices (including THER, rubber tube, headband, band) was significant (Supplementary Figures S7, S11) with the exception of lateral flexion (Supplementary Figures S15, S18). In addition, a larger neck training device called the multi-cervical unit (MCU) has also been used in pilot neck training (Chiu and Sing, 2002; Burnett et al., 2005). Burnett et al. (Burnett et al., 2005) compared the effectiveness of muscle strength training in pilots using MCU or THER. The researchers found that MCU was superior to elastic bands. Although our analysis included this study as a separate subgroup, we cannot conclude that MCU was more effective than elastic bands for neck muscle training due to the lack of additional literature support. Netto et al. (Netto et al., 2007) performed neck exercises in pilots using elastic bands and resistance machines to simulate different intensities of muscle activation during air combat maneuver. They concluded that neck training using the elastic band might be most practical for pilots who were exposed to low gravity flight and maintained a neutral neck position, such as transport, bomber, or helicopter pilots. In addition, elastic band training is more suitable for rehabilitation after + Gz injury. On the other hand, resistance machines were recommended for overload intensity training of fighter pilots to obtain maximal muscle strength (TAN, 1999; Coutts et al., 2007; Cheng et al., 2011).

Some studies applied virtual reality (VR) systems for training assistance and instruction (Bahat et al., 2020), but did not report differences in terms of muscle strength improvement and pain relief. Given that we do not know what devices their subjects used during training, it is not possible to categorize it into any of the subgroups. The frequent deployments and relocations of pilots prevent them from regularly accessing the gym for training and make it difficult to obtain training equipment suitable for counterweight (Rausch et al., 2021). Therefore, we need to seek forms of strength exercise that do not require special equipment, and the training equipment is portable (O'Conor et al., 2020). More studies suggested the use of an elastic band for training (Netto et al., 2007; Salmon et al., 2011) given its convenience compared to large equipment. One study reported a wearable cervical resistance exercise device that has been shown to be effective in improving strength and endurance in the cervical muscle. Targeted at implementing portable countermeasures, this device may represent a good alternative to elastic bands (O'Conor et al., 2020).

### 4.3.3 Training protocol

In general occupational groups, work-related neck pain is reduced by various physical exercises (Blangsted et al., 2008), proprioceptive muscle coordination training (Waling et al., 2000; Blomgren et al., 2018), and strength training (Lange et al., 2013; Price et al., 2020). Currently, there is no recognized form of exercise that can prevent neck pain in pilots. The most common form of training that target the neck and shoulder muscle include strength, endurance, and coordination training (Lange et al., 2013; Murray et al., 2017; Rausch et al., 2021). It has been reported that muscle strength training for more than 1 hour per week may represent a protective factor for neck pain in helicopter pilots (Ang et al., 2009). Many investigators have used electromyographic measures based on flight exposure to assess neck and shoulder fatigue and found prolonged muscle activation, indicating that muscle fatigue may be a risk factor for the development of neck and shoulder pain (Ang et al., 2005; Harrison et al., 2009; Salmon et al., 2011). In the general population, the strength and endurance of cervical muscles are decreased in patients with neck pain (Falla et al., 2004; Selistre et al., 2021). Hämäläinen et al, (1998) investigated the effects of a training program on pilots and found that dynamic endurance trainers had less sick leave and +Gz limitation due to neck complaints than resistance training. This finding suggests that pilots with neck and shoulder pain may also benefit from endurance training rather than just strength training. Generally, in muscle fibers, type I fibers are activated superior to type II fibers to provide sustained low-intensity muscle pull (Visser and van Dieën, 2006). Increasing the number of type I fibers can increase the ability of muscles to aerobically breathe, maintaining muscle contraction against fatigue for extended periods of time (Harrison et al., 2009). A longer duration of pain is associated with significantly reduced number of type I fibers and higher proportion type II fibers (Purushotham et al., 2022). Endurance training programs that provide low loads have the potential to slow or reverse this change.

In addition to endurance training, an increasing number of studies emphasize the importance of pilot coordination training (Salmon et al., 2013; Lange et al., 2014; Murray et al., 2020; Rausch et al., 2021). Superficial musculature is used for segmental control during neck movements, which requires deep stability to support the anterior convex curve of the cervical spine (Salmon et al., 2011). When the deep muscles are weakened, the superficial muscles overcompensate, leading to dysfunction of the neck muscles (Falla, 2004). Falla, (2004) proposed the use of low-load exercise to re-establish coordination between the deep and superficial layers of the neck muscles. Salmon et al, (2013) performed neck coordination and endurance training on pilots and showed that the participants in both groups exhibited significant increases in maximum neck strength and endurance compared to the control group, and the effect of the coordination training group showed a greater increase than the endurance training group. In another study, pilots used trampoline training to improve neck muscle function, and the results showed that it was as effective as strength training in increasing maximal muscle strength and reducing neck strain in flight. Trampoline training may increase neuromuscular performance and intermuscular coordination, which may improve the mechanical efficiency of maintaining cervical spine stability and thus have beneficial effects in reducing flight muscle strain (Sovelius et al., 2006). Our subgroup analysis showed that strength training alone did not improve the neck muscle strength of pilots (Supplementary Figures S8, S12, S16, S19) or reduce the prevalence of neck pain (Figure 6), In contrast, strength with endurance training significantly improved neck muscle strength (Supplementary Figures S8, S12, S16, S19) but also did not show a significant reduction in the prevalence of neck pain. In contrast, a combination of strength, endurance and coordination training appeared to achieve significant neck pain relief. However, due to the small pooled number, we need to include more studies to verify this finding.

#### 4.3.4 Follow-up period

In the subgroup analysis of follow-up time, muscle strength was improved in the follow-up less than 20 weeks, while the follow-up group with no less than 20 weeks had a significant improvement in the extension but not in the flexion muscle. (Supplementary Figures S9, S13). However, we generally believe that the exercise period can accumulate higher changes in outcomes. However, the length of the training period greatly affects compliance. Ang et al. (Ang et al., 2009) reported 77% compliance at a follow-up time of 6 weeks, which was acceptable. Another study conducted a 12-weeks training follow-up and reported 52.8% and 76.1% adherence in the two groups (Salmon et al., 2013). The study by Murray et al. (Murray et al., 2017; Murray et al., 2020) included a long follow-up time (20 weeks), whereas only 28.6% of participants adhered to regular training. In the study by Lange et al. (Lange et al., 2014) with a follow-up of 24 weeks, only 58% of the participants regularly trained three times a week. Overall, the compliance of pilots is reduced with a longer follow-up period.

Therefore, we need to identify the optimal training period to achieve the maximum payoff. Bahat et al, (2020) reported self-supervised 4-weeks short-period training in pilots, but the results showed no significant improvement in muscle strength. Other groups reported that 6 weeks of training increased muscle strength (Stasinaki et al., 2015) and reduced in-flight neck strain or pain under + Gz loading (Sovelius et al., 2006). For office workers, their neck pain was reduced when they performed specific strength training for 10 weeks (Saeterbakken et al., 2017). Similarly, Burnett et al. (Burnett et al., 2005) suggested neck training 10 weeks before pilots start flying high-performance aircraft to be completely prepared for the load of high-performance. In addition, a neck strengthening program should be developed for those who return to service after a flight break to increase neck strength. Based on previous research experience, the research and development of exercise programs for future combat pilots should include exercise schedules and supervised programs to ensure compliance (Bahat et al., 2020). Based on this notion, we believe that a short period of adequate stimulation is more suitable than a longer exercise program for the professional needs of pilots, and this time can be roughly limited to approximately 6–12 weeks. Moreover, training must be highly specific and supervised because general whole-body reinforcement is not thought to produce similar improvements in neck strength (Conley et al., 1997). In the future, an informatization management means that breaks geographical restrictions are indispensable.

### 4.4 Limitations

This study has some limitations. First, we included observational studies (cohort studies, case-control studies, and cross-sectional studies), when investigating the effects of exercise on neck pain. This design will cause an unavoidable bias due to the presence of potential confounding factors. Second, the number of studies included in each outcome was small, and the quality was uneven. The small sample size of each study leads to a wide confidence interval for the pooled effect value, which reduces the power of the results. Third, differences in the definition of outcomes and measurement criteria were noted. There were differences in the criteria for the pain and pain-free groups between studies. Fourth, differences in exercise programs were noted. To address the above limitations, we will continuously follow up the relevant research progress and try to identify more high-quality studies to expand the sample size. The mesh meta-analysis will be performed to compare the differences between types of exercise intervention.

## 5 Conclusion

Physical exercise can improve the neck muscle strength of military pilots and is significantly effective in flexion, extension, and left and right lateral flexion. Moreover, fighter pilots, complex devices, comprehensive training (strength plus endurance), and a follow-up period less than 20 weeks seemed to obtain more significant muscle strength improvement than helicopter pilots, small devices, simple strength training, and a follow-up period greater than 20 weeks. Overall, the pooled results did not show a significant effect of exercise on neck pain. However, sensitivity analysis revealed that the lack of a significant effect was due to heterogeneity. The sources of heterogeneity may include observational studies and studies with small samples. After removing the above studies, exercise showed a significant protective effect on neck pain. No significant differences in shoulder muscle strength or neck and shoulder pain intensity were noted between exercises. However, this conclusion should be taken with caution, and more studies need to be included to improve the persuasiveness of these findings. There are great challenges in the development of training programs for military pilots due to the differences in aircraft types and the uncertainty of working hours and locations. In the future, training protocols, equipment, periods and methods of supervision should be fully considered.

## Data Availability

The raw data supporting the conclusion of this article will be made available by the authors, without undue reservation.
