# Supplementary material for: Physical exercise improved muscle strength and pain on neck and shoulder in military pilots
Source: Front Physiol. 2022 Sep 2;13:973304. doi: 10.3389/fphys.2022.973304 (PMC9479108; doi:10.3389/fphys.2022.973304)
Supplement: Supplementary file 1 [file DataSheet1.docx]

Supplementary Material

# Supplementary Tables

| **Supplementary Table 1. Search strategy** | |
| --- | --- |
| Database | Search strategy |
| Pubmed | #1 "pilot"[Title/Abstract] OR "pilots"[Title/Abstract] OR "Co-Pilot"[Title/Abstract] OR "Co-Pilots"[Title/Abstract] OR "aircrew"[Title/Abstract] OR "aviator"[Title/Abstract] OR "aviators"[Title/Abstract]  #2 "Exercise"[Title/Abstract] OR "Exercises"[Title/Abstract] OR "physical activity"[Title/Abstract] OR "activities physical"[Title/Abstract] OR "activity physical"[Title/Abstract] OR "physical activities"[Title/Abstract] OR "exercise physical"[Title/Abstract] OR "exercises physical"[Title/Abstract] OR "physical exercise"[Title/Abstract] OR "physical exercises"[Title/Abstract] OR "exercise isometric"[Title/Abstract] OR "exercises isometric"[Title/Abstract] OR "isometric exercises"[Title/Abstract] OR "isometric exercise"[Title/Abstract] OR "exercise training"[Title/Abstract] OR "exercise trainings"[Title/Abstract] OR "training exercise"[Title/Abstract] OR (("education"[MeSH Subheading] OR "education"[All Fields] OR "Training"[All Fields] OR "education"[MeSH Terms] OR "train"[All Fields] OR "train s"[All Fields] OR "trained"[All Fields] OR "training s"[All Fields] OR "Trainings"[All Fields] OR "trains"[All Fields]) AND "Exercise"[Title/Abstract]) OR "Training"[Title/Abstract] OR "resistance training"[Title/Abstract] OR "training resistance"[Title/Abstract] OR "strength training"[Title/Abstract] OR "training strength"[Title/Abstract] OR "endurance training"[Title/Abstract] OR "training endurance"[Title/Abstract]  #3 "neck"[Title/Abstract] OR "Shoulder"[Title/Abstract]  #4 #1 AND #2 AND #3 |
| Embase | #1. 'pilot'/exp OR 'pilot':ab,ti OR 'pilots':ab,ti OR 'co-pilot':ab,ti OR 'co-pilots':ab,ti OR 'aircrew':ab,ti OR 'aviator':ab,ti OR 'aviators':ab,ti  #2. 'exercise'/exp OR 'exercise':ab,ti OR 'exercises':ab,ti OR 'physical activity':ab,ti OR 'activities, physical':ab,ti OR 'activity, physical':ab,ti OR 'physical activities':ab,ti OR 'exercise, physical':ab,ti OR 'exercises, physical':ab,ti OR 'physical exercise':ab,ti OR 'physical exercises':ab,ti OR 'exercise, isometric':ab,ti OR 'exercises, isometric':ab,ti OR 'isometric exercises':ab,ti OR 'isometric exercise':ab,ti OR 'exercise training':ab,ti OR 'exercise trainings':ab,ti OR 'training, exercise':ab,ti OR 'trainings, exercise':ab,ti OR 'training'/exp OR 'resistance training':ab,ti OR 'training, resistance':ab,ti OR 'strength training':ab,ti OR 'training':ab,ti OR 'training, strength':ab,ti OR 'endurance training':ab,ti OR 'training, endurance':ab,ti  #3 'neck':ab,ti OR 'shoulder':ab,ti  #4 #1 AND #2 AND #3 |
| Cochrane library | #1 (pilot):ti,ab,kw OR (pilots):ti,ab,kw OR (Co-Pilot):ti,ab,kw OR (Co-Pilots):ti,ab,kw OR (aircrew):ti,ab,kw  #2 (aviators):ti,ab,kw OR (aviators):ti,ab,kw  #3 (Exercise):ti,ab,kw OR (Physical Activity):ti,ab,kw OR (Activities, Physical):ti,ab,kw OR (Activity, Physical):ti,ab,kw OR (Physical Activities):ti,ab,kw  #4 (Exercise, Physical):ti,ab,kw OR (Exercises, Physical):ti,ab,kw OR (Physical Exercise):ti,ab,kw OR (Physical Exercises):ti,ab,kw OR (Exercise, Isometric):ti,ab,kw  #5 (Exercises, Isometric):ti,ab,kw OR (Isometric Exercises):ti,ab,kw OR (Isometric Exercise):ti,ab,kw OR (Exercise Training):ti,ab,kw OR (Exercise Trainings):ti,ab,kw  #6 (Training, Exercise):ti,ab,kw OR (Trainings, Exercise):ti,ab,kw OR (Training):ti,ab,kw OR (Resistance Training):ti,ab,kw OR (Training, Resistance):ti,ab,kw  #7 (Strength Training):ti,ab,kw OR (Training, Strength):ti,ab,kw OR (Endurance Training):ti,ab,kw OR (Training, Enduranc):ti,ab,kw  #8 (Neck):ti,ab,kw OR (Shoulder):ti,ab,kw  #9 #1 or #2  #10 #3 or #4 or #5 or #6 or #7  #11 #8 and #9 and #10 |

| **Supplementary Table 2. Inclusion/exclusion criteria of literature** | | |
| --- | --- | --- |
| **PICOS** | **Inclusion** | **Exclusion** |
| P | Professional pilots driving any aircraft were included in our study | Flight trainees who have not flown. |
| I | 1. In randomized controlled trials (RCTs), subjects in the experimental group received a certain intensity, regular physical training or exercise, including strength, endurance or coordination 2. In a retrospective study, subjects in the exposure group received a certain period of regular physical training or exercise including strength, endurance or coordination 3. No limit on sample size | 1. Other types of exercises, such as aerobic training, nonphysical exercise, etc. 2. Irregular training |
| C | Pilots who do not received any physical training or only underwent routine training | 1. No control group 2. Control group were nonpilots |
| O | 1. Neck or shoulder muscle strength 2. Prevalence of neck or shoulder pain 3. Intensity of neck or shoulder pain | Relevant outcomes were missing |
| S | RCTs or observational studies | Case reports, abstracts, letters, commentaries and reviews |

| **Supplementary Table 3. Scores of the Newcastle-Ottawa Quality Assessment Scale for Observational Study.** | | | | |
| --- | --- | --- | --- | --- |
|  | Selection | Comparability | Exposure | Total score |
| Hämäläinen  1993 | ★★ | - | ★★ | ★★★★ |
| Newman  1997 | ★★★ | ★ | ★ | ★★★★★ |
| Albano  1998 | ★★★ | ★ | ★ | ★★★★★ |
| De Loose  2008 | ★★★★ | - | ★★ | ★★★★★★ |

| **Supplementary Table 4. Characteristics of the included studies** | | | | | | | | | | | |
| --- | --- | --- | --- | --- | --- | --- | --- | --- | --- | --- | --- |
| Study | Country | Type of aircraft | N | Age (years) | Height (m) | Weight (kg) | Type of study | Training protocol | Equipment | Follow-up period | Outcome |
| Hämäläinen, 1993 | Finland | Fighter | 27 | NA | NA | NA | OS | Neck endurance training | NA | NA | Incidence of acute inflight neck pain |
| Newman, 1997 | Australia | Fighter | 40 | Total: 29.6±6.5 | Total: 181±5.75 | Total: 81±8.25 | OS | Neck strengthening exercise | NA | NA | Frequency of neck pain |
| Albano, 1998 | USA | Fighter | 229 | Total: 35.3±4.9 | Total: 179.8±6.4 | Total: 81.3±8.5 | OS | Neck strength exercise, for 2.3 times/wk | Neck machine and freehand | NA | Prevalence of neck pain (injury) |
| Jones, 2000 | USA | Fighter | 66 | NA | NA | NA | OS | Regular strength training for the neck muscles. | NA | NA | Incidence of in-flight or post-flight pain episodes |
| Alricsson, 2004 | Sweden | Fighter | 40 | TG: 29.4±4.5 CG: 29.4±3.1 | TG: 1.82±5.2 CG: 1.80±5.1 | TG: 81.1±8.0 CG: 80.0±6.8 | RCT | Neck strength and endurance exercises, for 3 times/wk | Rubber tube and weights attached | 6-8 mo | Maximum isometric strength (flexion, extension) |
| Burnett, 2005 (A) | Australia | Fighter | 23 | TG: 23.3±4.0 CG: 22.6±4.4 | TG: 1.82±4.0 CG: 1.82±4.3 | TG: 78.8±13.2 CG: 76.4±7.3 | RCT | Based upon an intensive-interval neck strength-endurance model, for 2x30min/wk | Multi-cervical unit (MCU) | 10 wk | Maximum isometric strength (flexion, extension, Ltflx and Rtflx) |
| Burnett, 2005 (B) |  |  | 20 | TG: 21.7±3.1 CG: 22.6±4.4 | TG: 1.81±7.2 CG: 1.82±4.3 | TG: 75.8±13.6 CG: 76.4±7.3 |  |  | Thera-Band tubing |  |  |
| De Loose, 2008 | Belgium | Fighter | 90 | NA | NA | NA | OS | Neck strength training | NA | NA | Prevalence of pain |
| Ang, 2009 | Sweden | Helicopter | 68 | TG: 37.3±6.4 CG: 37.7±5.4 | TG: 181±4 CG: 182±6 | TG: 81.0±6.3 CG: 82.6±9.9 | RCT | Neck endurance-strength exercises, for once to twice daily, 10 to 15 min/session | Elastic rubber bands | 6 wk | Prevalence of neck pain |
| Salmon, 2013 (A) | Canada | Helicopter | 18 | TG: 37.18±4.5 CG: 37.12±6.31 | TG: 1.80±0.08 CG: 1.79±0.07 | TG: 86.03±12.28 CG: 90.05±11.24 | RCT | Coordination exercises focused on muscle control through three stages, for 3 times/wk | Freehand and elastic rubber tubing | 12 wk | Isometric maximal voluntary contraction (flexion, extension, Ltflx and Rtflx) |
| Salmon, 2013 (B) |  |  | 19 | TG: 35.40±8.22 CG: 37.12±6.31 | TG: 1.74±0.077 CG: 1.79±0.07 | TG: 77.33±24.07 CG: 90.05±11.24 |  | Endurance by resisting the dynamic movements of cervical, for 3 times/wk | Elastic rubber tubing |  |  |
| Lange, 2013 | Denmark | Fighter | 54 | TG: 31±5.2 CG: 33.5±4.8 | TG: 181±3.0 CG: 182±5.2 | TG: 79±7.4 CG: 79±10.4 | RCT | Neck and shoulder strength, endurance, and coordination training, for 3x20min/wk | Dumbbells, bodyblade, and headband | 24 wk, | Prevalence of neck pain; Pain intensity on a scale 0 to 10 |
| Lange, 2014 | Denmark | Fighter | 55 | NA | NA | NA | RCT | Neck strength, endurance, and coordination training, for 3x20min/wk | Dumbbells, bodyblade, and headband | 24 wk | MVC (shoulder elevation) |
| Murray, 2017 | Denmark | Helicopter | 44 | TG: 40.4±6.7 CG: 40.7±8.4 | TG: 182±7 CG: 180±8 | TG: 84.2±12.7 CG: 83.7±11.8 | RCT | Strength, endurance, and coordination training targeting the neck and shoulder muscles, for 3x20min/wk | Elastic training bands and lightweight equipment. | 20 wk | Intensity of neck pain was assessed on a scale from 0-10 |
| Murray, 2020 | Denmark | Helicopter | 69 | TG: 40.4±6.7 CG: 40.7±8.4 | TG: 1.82±0.07 CG: 1.80±0.08 | TG: 84.2±12.7 CG: 83.7±11.8 | RCT | Neck and shoulder strength, endurance, and coordination training, for 3x20min/wk | Elastic training bands | 20 wk | MVC)(flexion, extension, shoulder elevation) |
| Bahat, 2020 | Israel | Fighter+Helicopter | 45 | TG: 30±5.8 CG: 28±5.1 | NA | NA | RCT | Self-kinematic neck strength training program, for 4x5min/wk | Equipment and VR system | 4 wk | Cervical isometric strength (flexion, extension); VAS |
| Rausch, 2021 | Germany | Helicopter | 18 | TG: 31±11 CG: 30±8 | NA | NA | RCT | Neck strength training and core stability phase, for 3x60min/wk | Sandbag, medicine  balls, bands, and small weights | 12 wk | Maximum isometric strength (flexion, extension, Ltflx and Rtflx) |
| NA=Not available; TG=Training group; CG=Control group; OS=Observational study; RCT=Randomized controlled trial; MVC=Maximal voluntary contraction; VAS=Visual analog scale; Ltflx=Left lateral flexion; Rtflx=Right lateral flexion | | | | | | | | | | | |

# Supplementary Figures


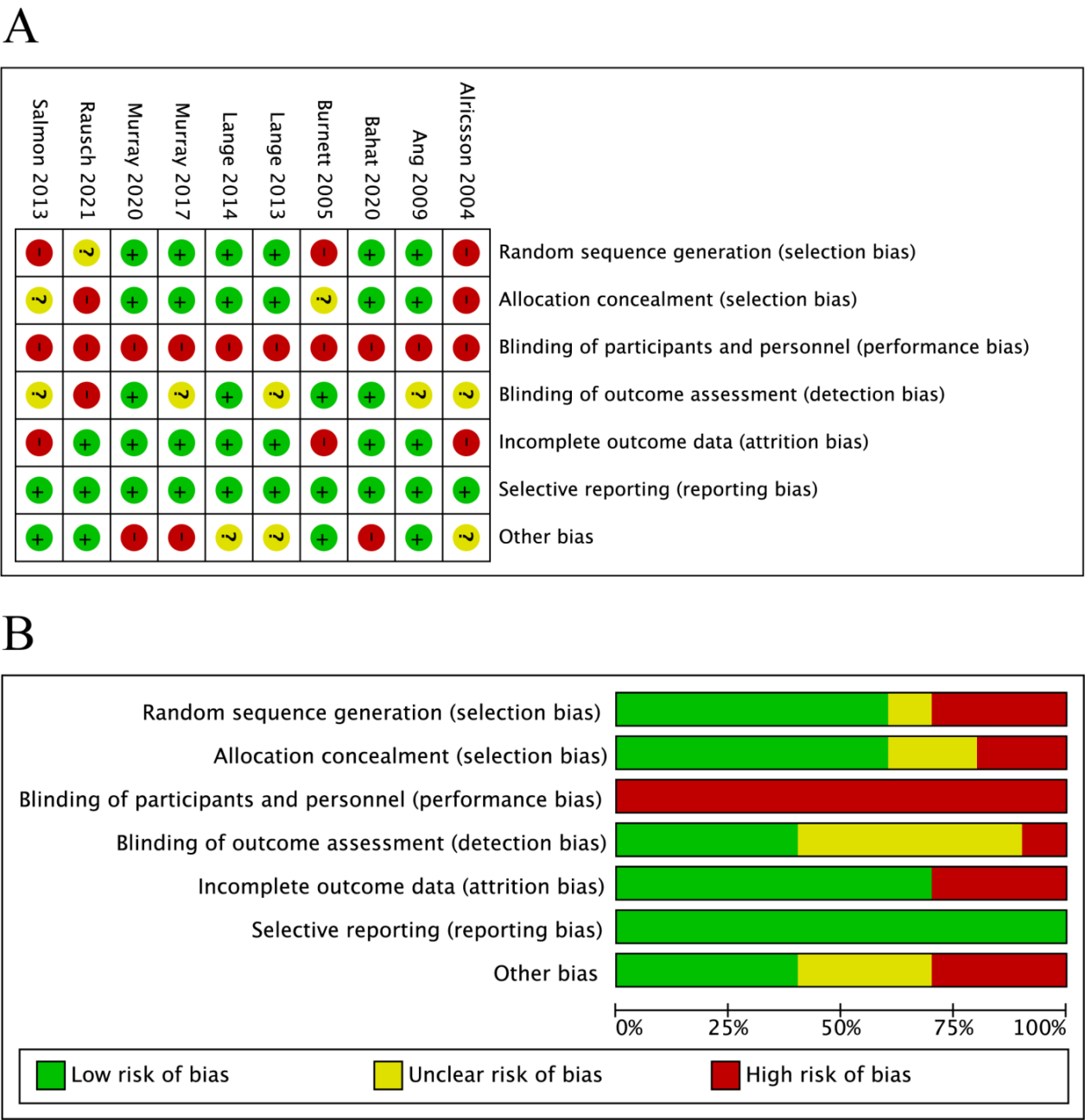


**Supplementary Figure 1.** **(A)** Risk of bias summary of included studies; **(B)** Risk of bias of included studies.


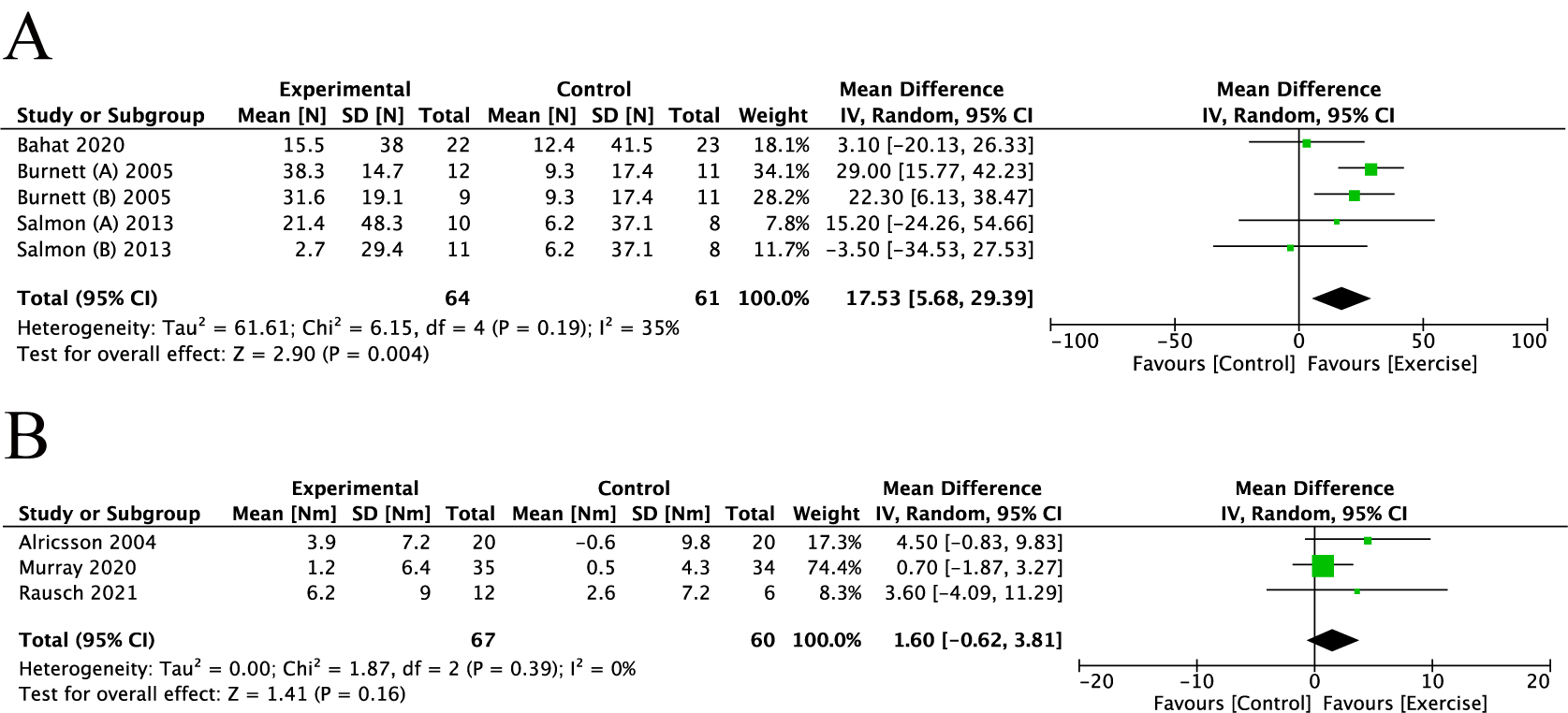


**Supplementary Figure 2.** The MVIC of the neck flexion in the unified unit. **(A)** N; **(B)** Nm.


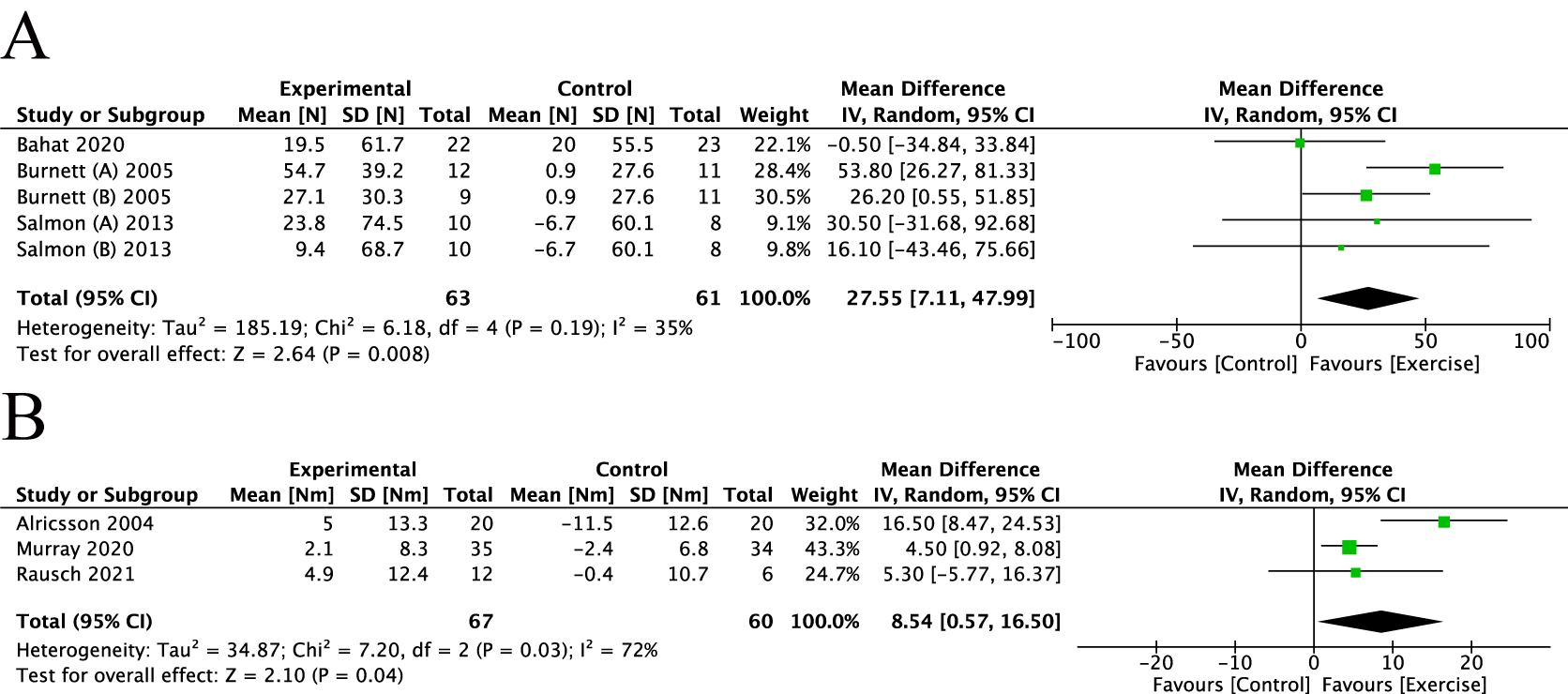


**Supplementary Figure 3.** The MVIC of the neck extension in the unified unit. **(A)** N; **(B)** Nm.


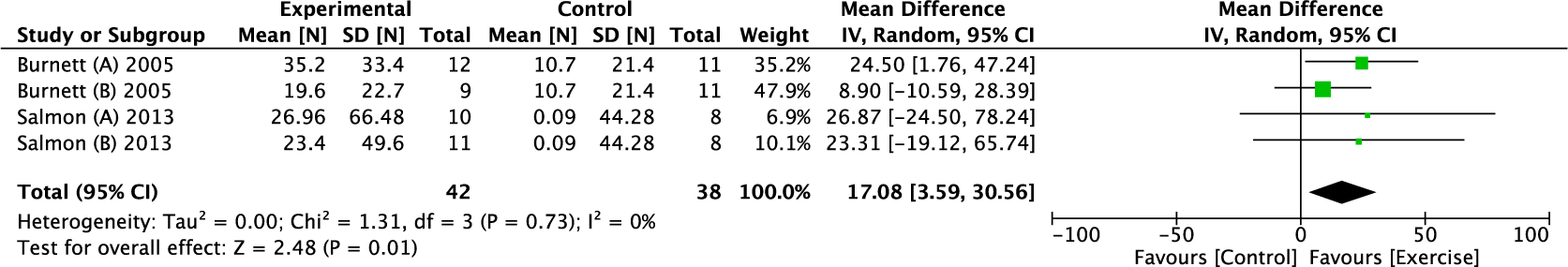


**Supplementary Figure 4.** The MVIC of the neck Rtflx in the unified unit.


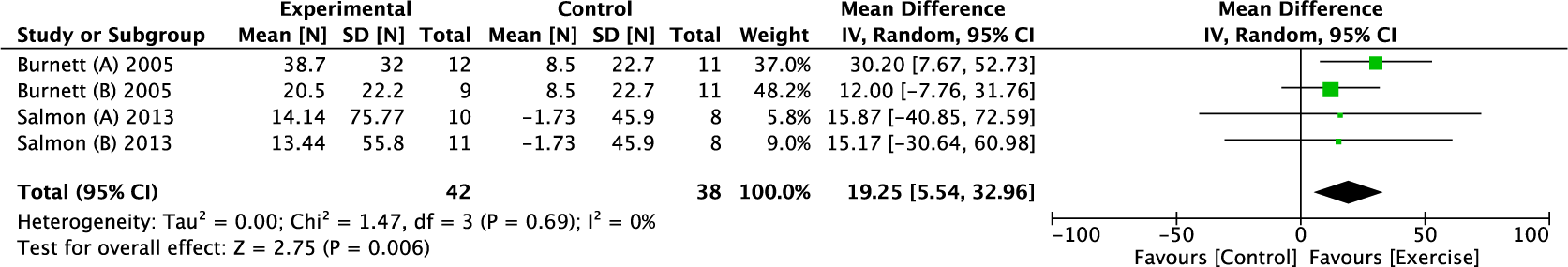


**Supplementary Figure 5.** The MVIC of the neck Ltflx in the unified unit.


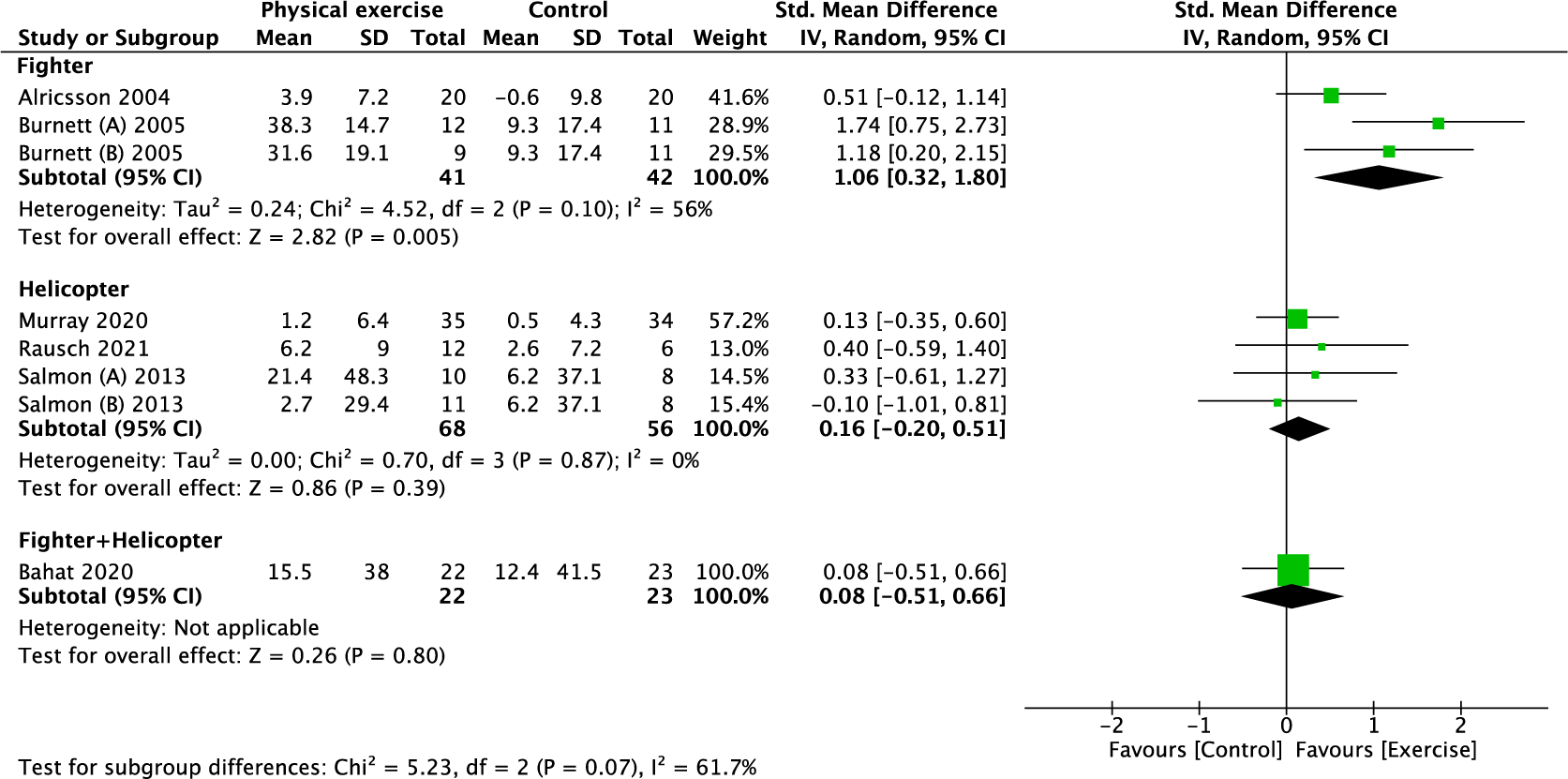


**Supplementary Figure 6.** Subgroup analysis for the MVIC of the neck flexion (type of aircraft).


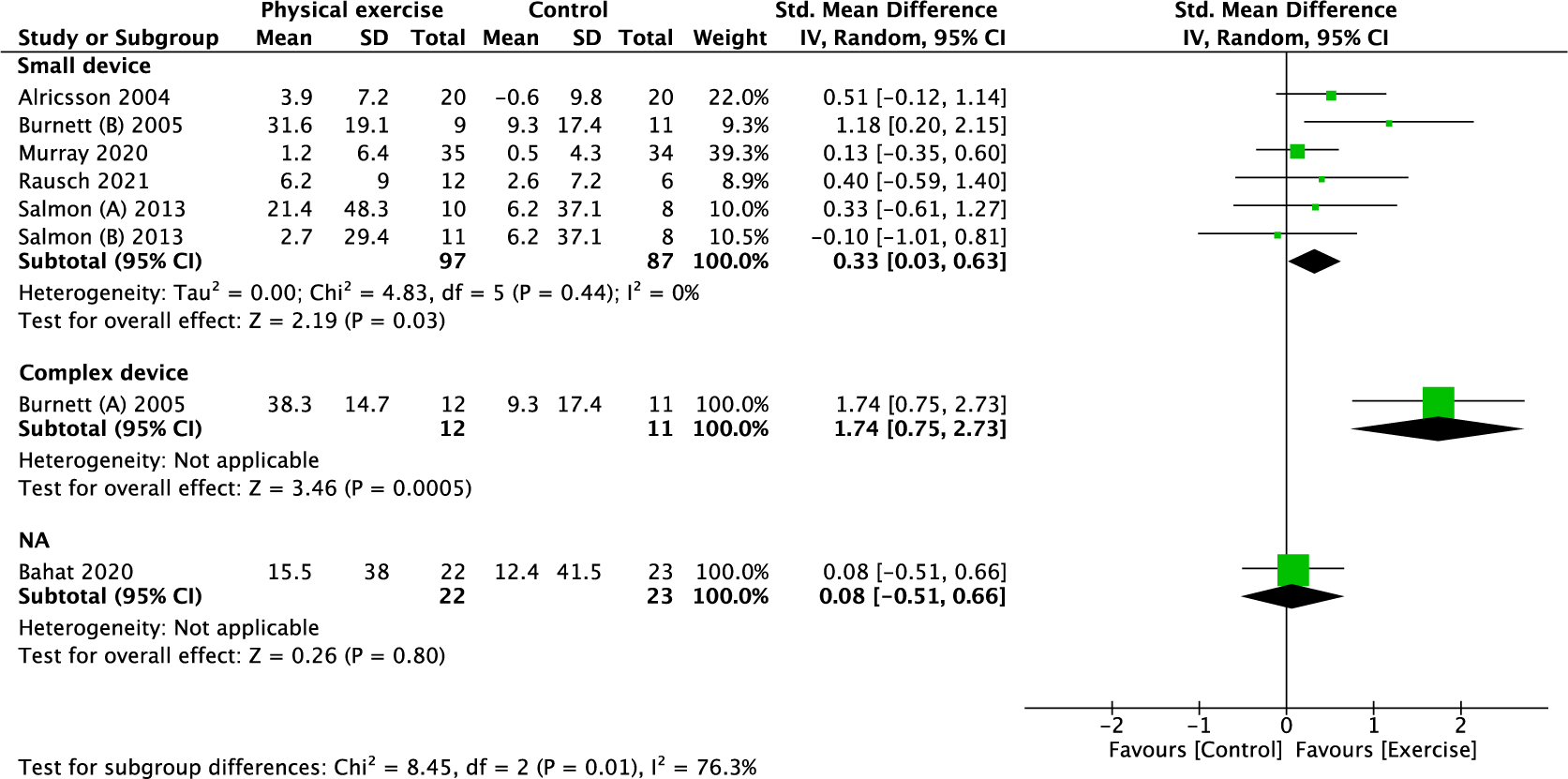


**Supplementary Figure 7.** Subgroup analysis for the MVIC of the neck flexion (equipment).


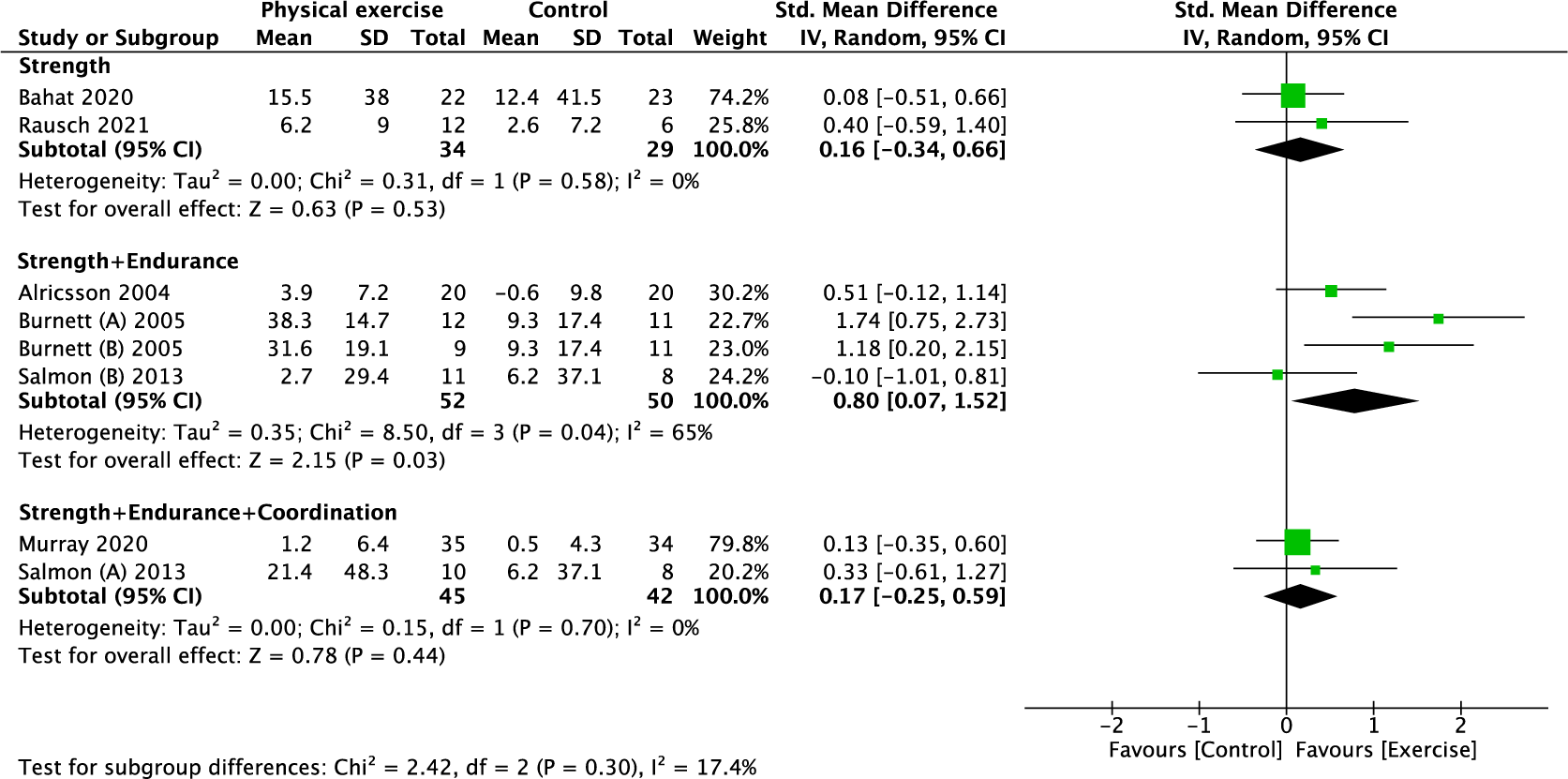


**Supplementary Figure 8.** Subgroup analysis for the MVIC of the neck flexion (training protocol).


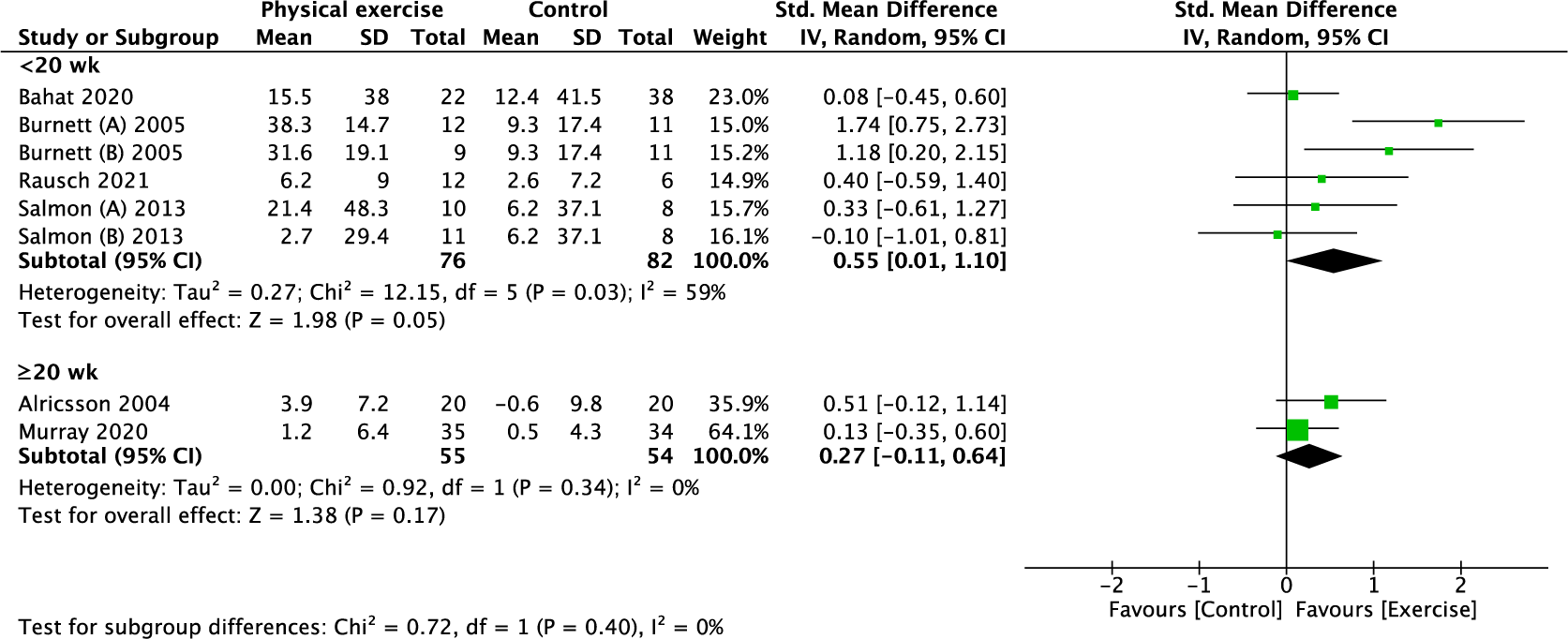


**Supplementary Figure. 9.** Subgroup analysis for the MVIC of the neck flexion (follow-up period).


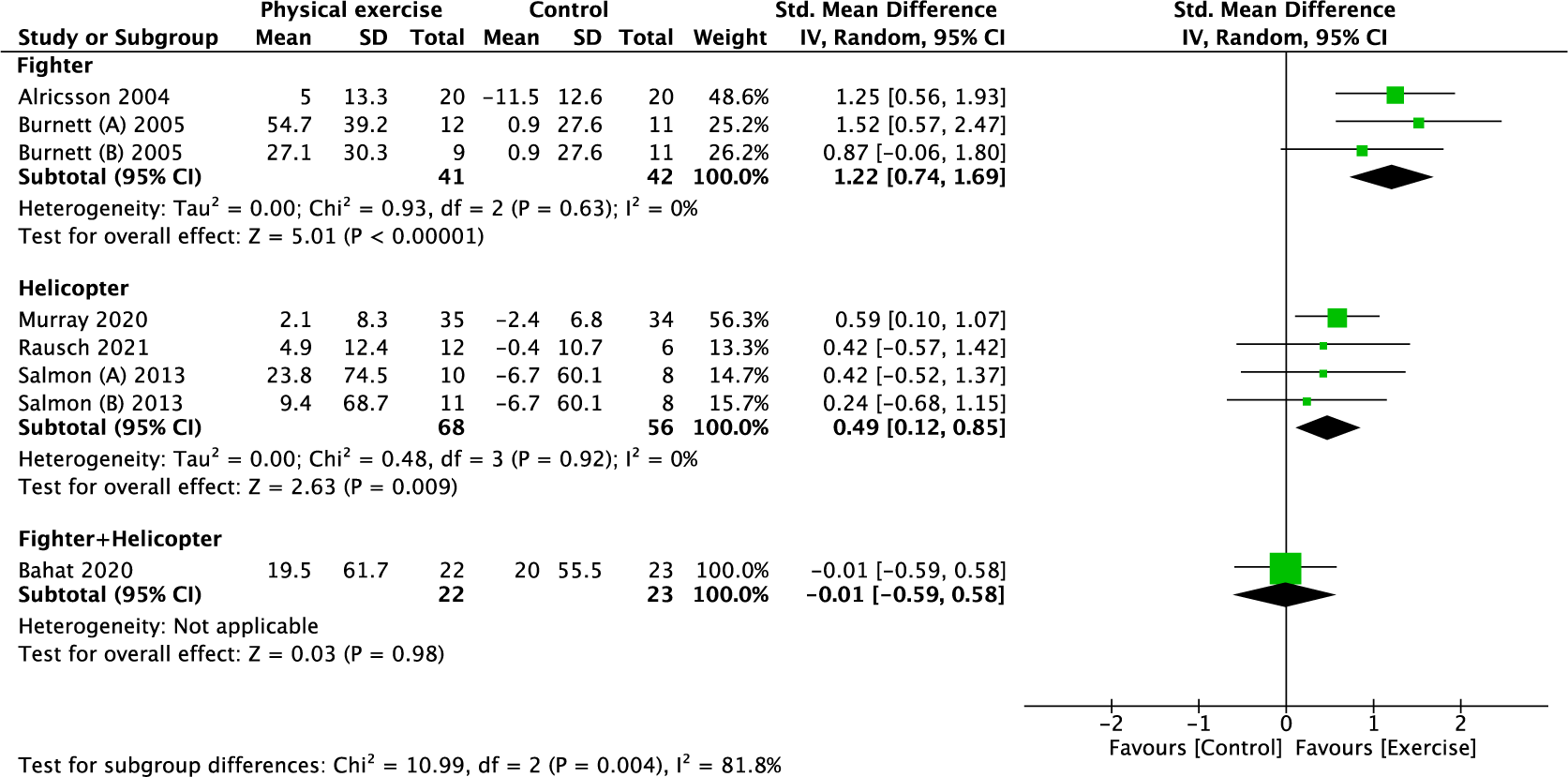


**Supplementary Figure 10.** Subgroup analysis for the MVIC of the neck extension (type of aircraft).


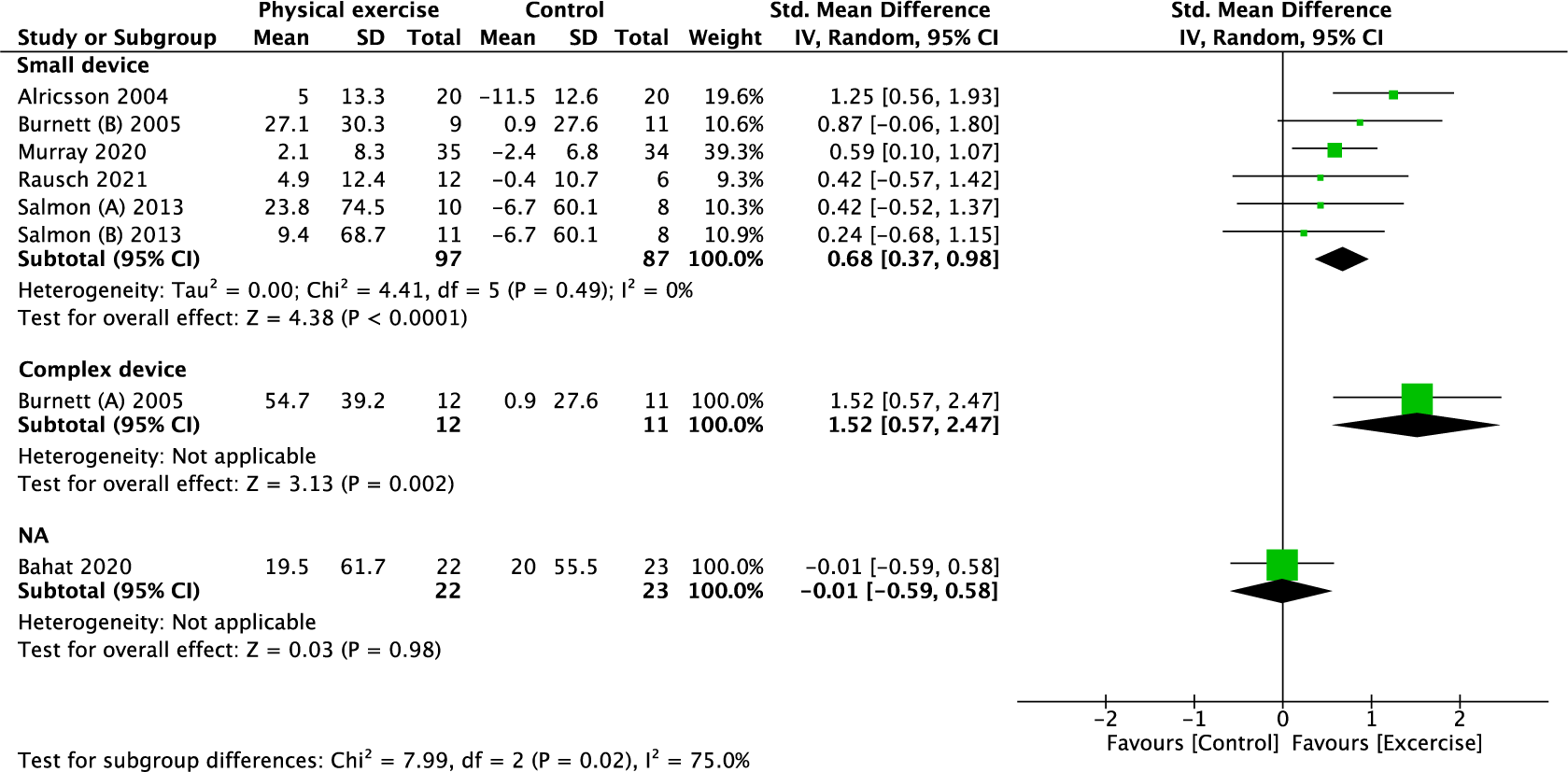


**Supplementary Figure 11.** Subgroup analysis for the MVIC of the neck extension (equipment).


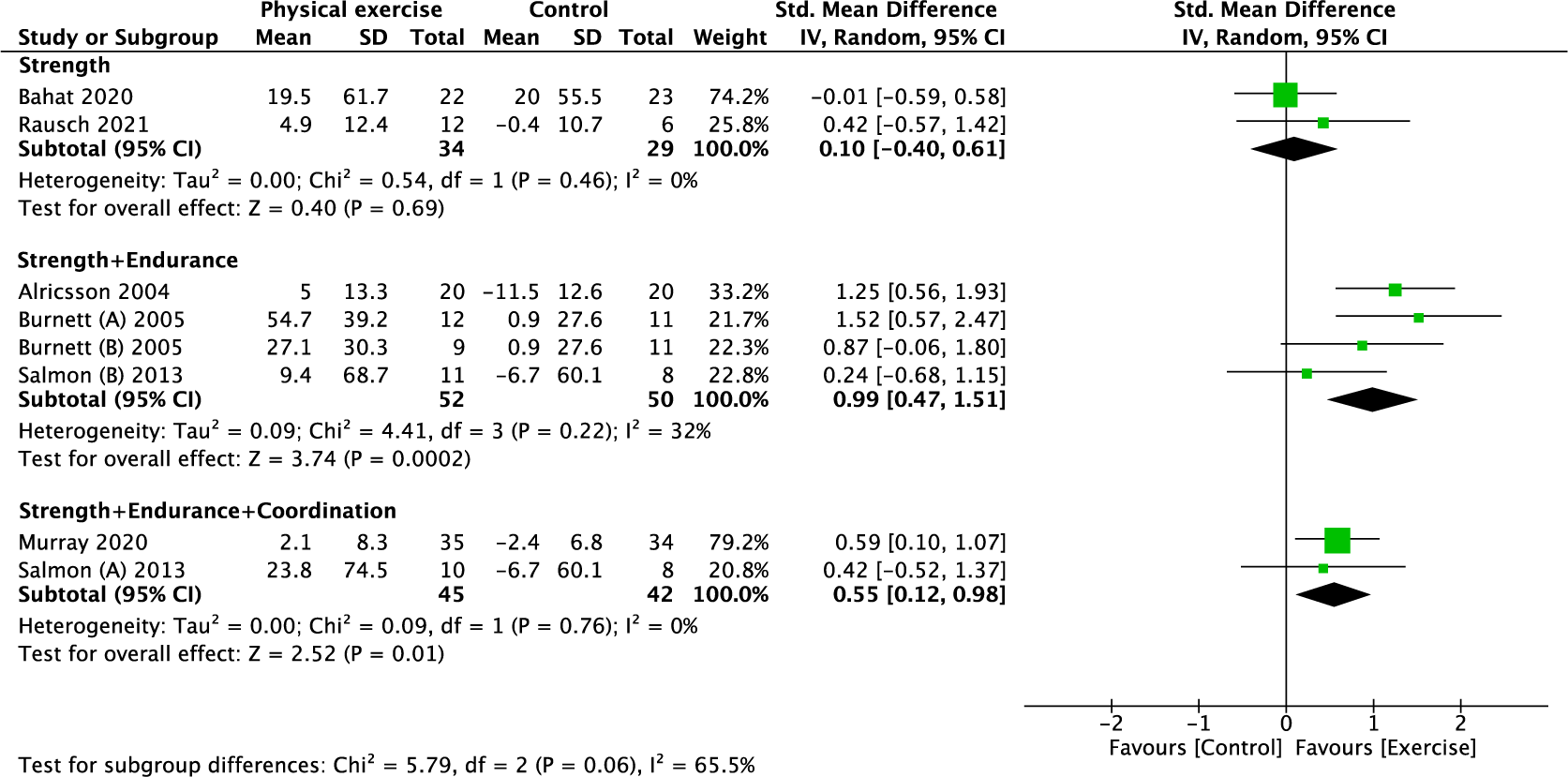


**Supplementary Figure 12.** Subgroup analysis for the MVIC of the neck extension (training protocol).


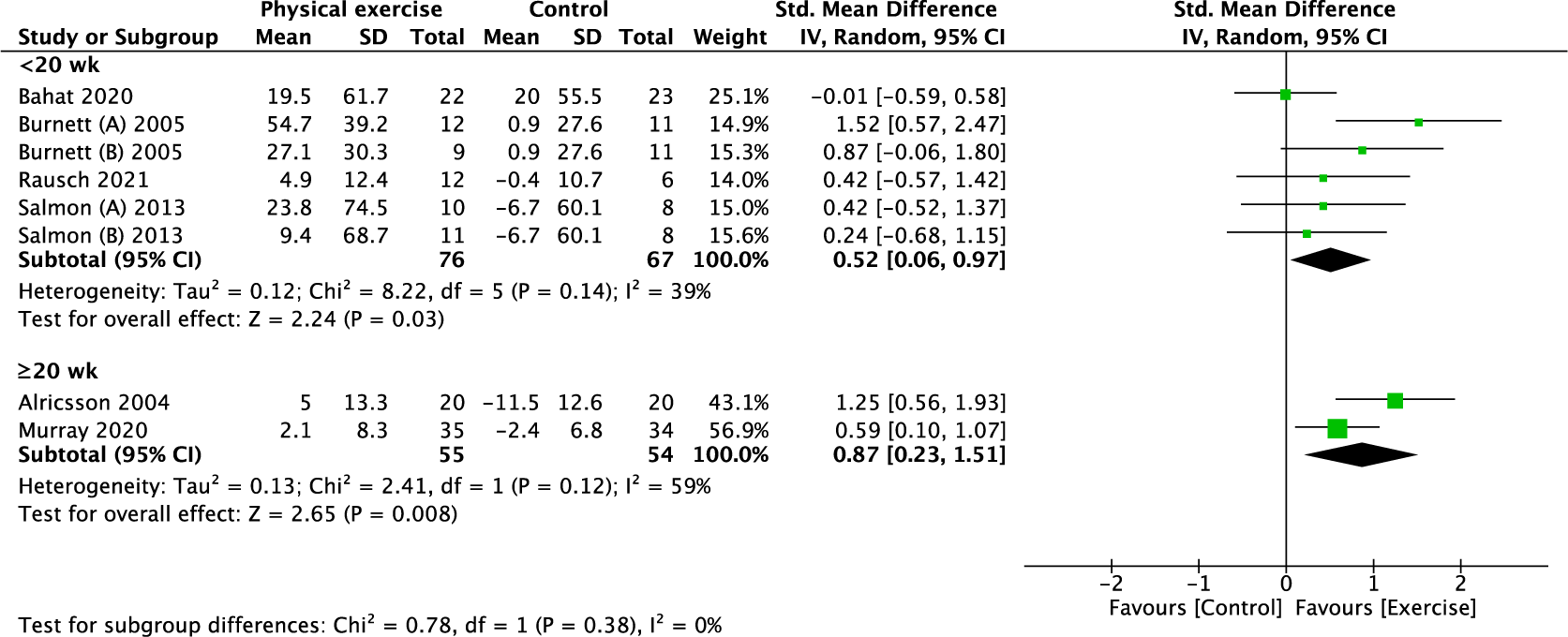


**Supplementary Figure 13.** Subgroup analysis for the MVIC of the neck extension (follow-up period).


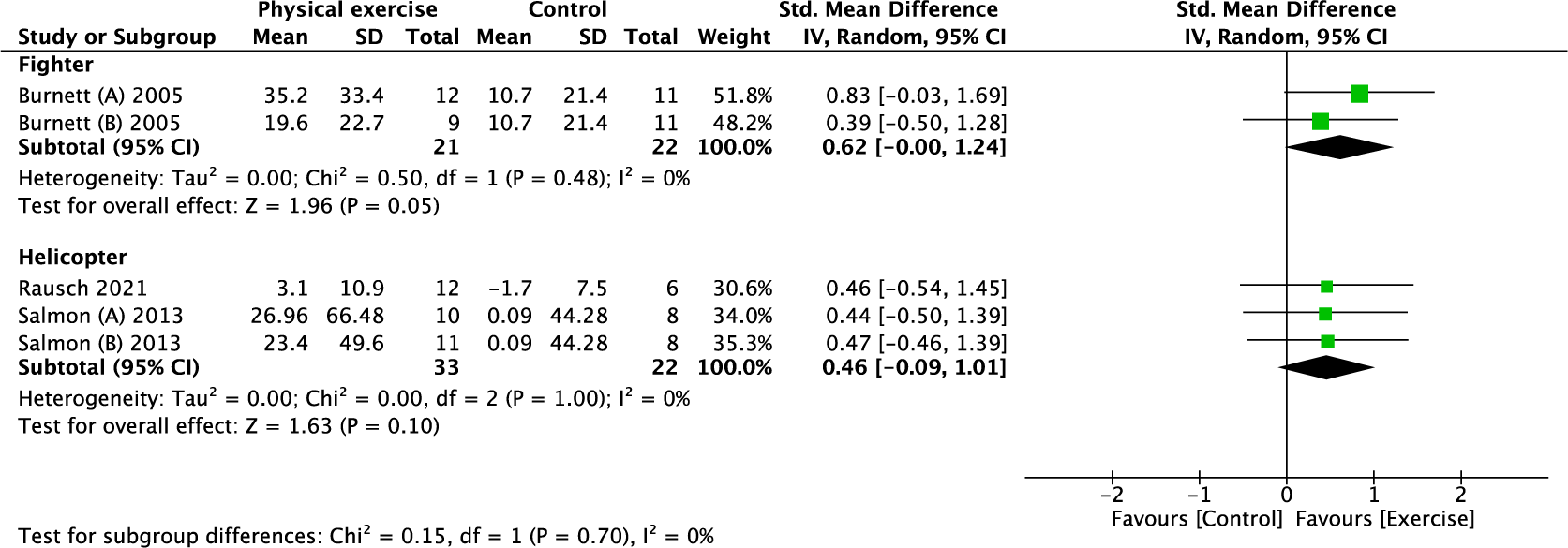


**Supplementary Figure 14.** Subgroup analysis for the MVIC of the neck Rtflx (type of aircraft).


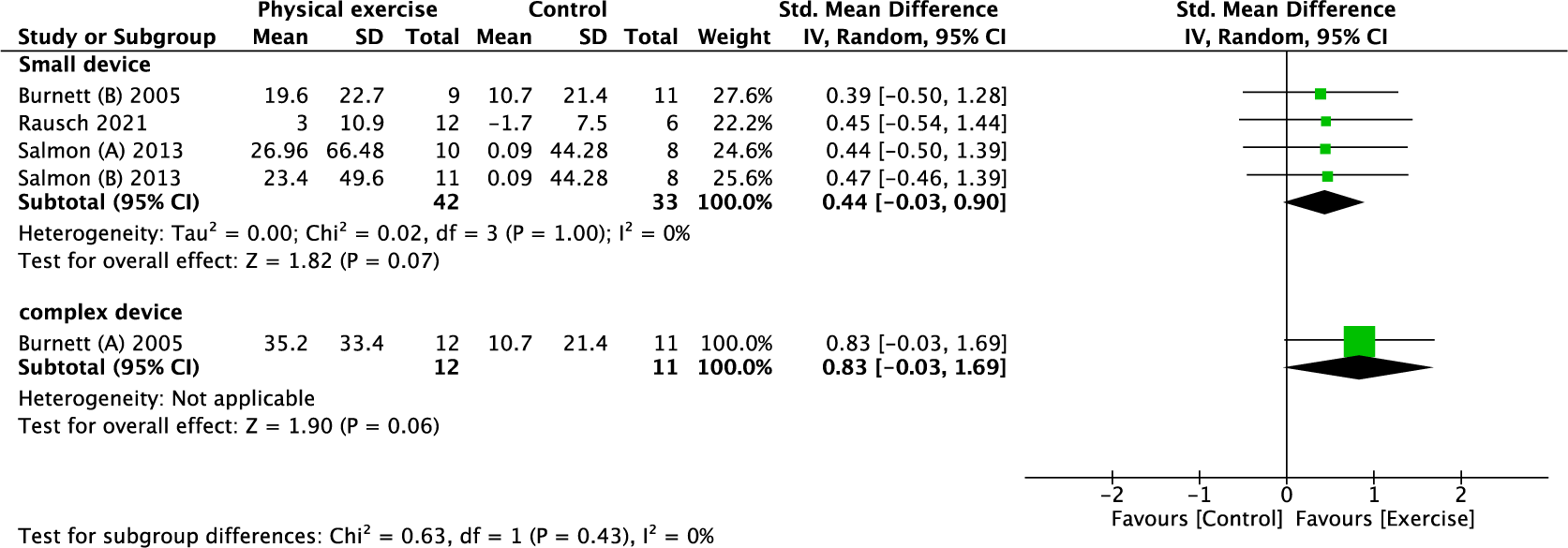


**Supplementary Figure 15.** Subgroup analysis for the MVIC of the neck Rtflx (equipment).


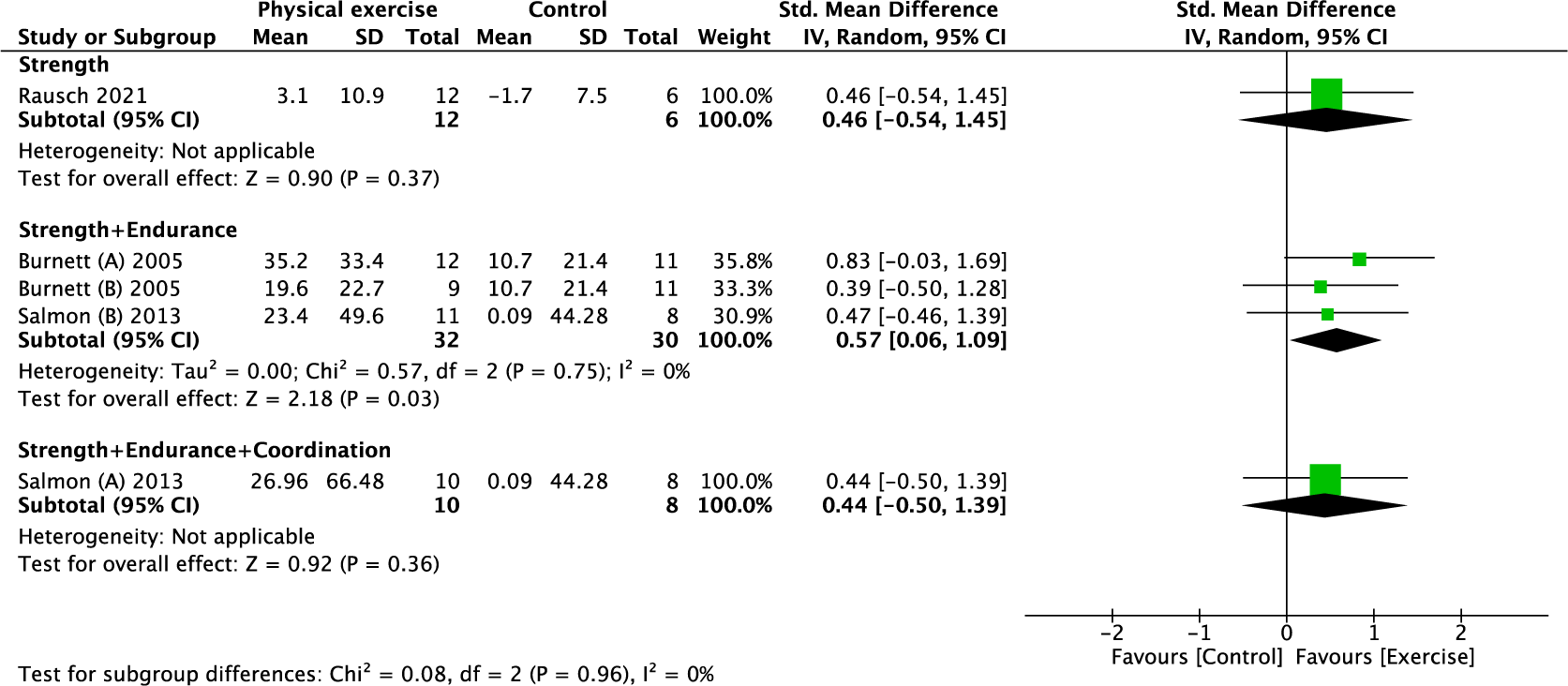


**Supplementary Figure 16.** Subgroup analysis for the MVIC of the neck Rtflx (training protocol).


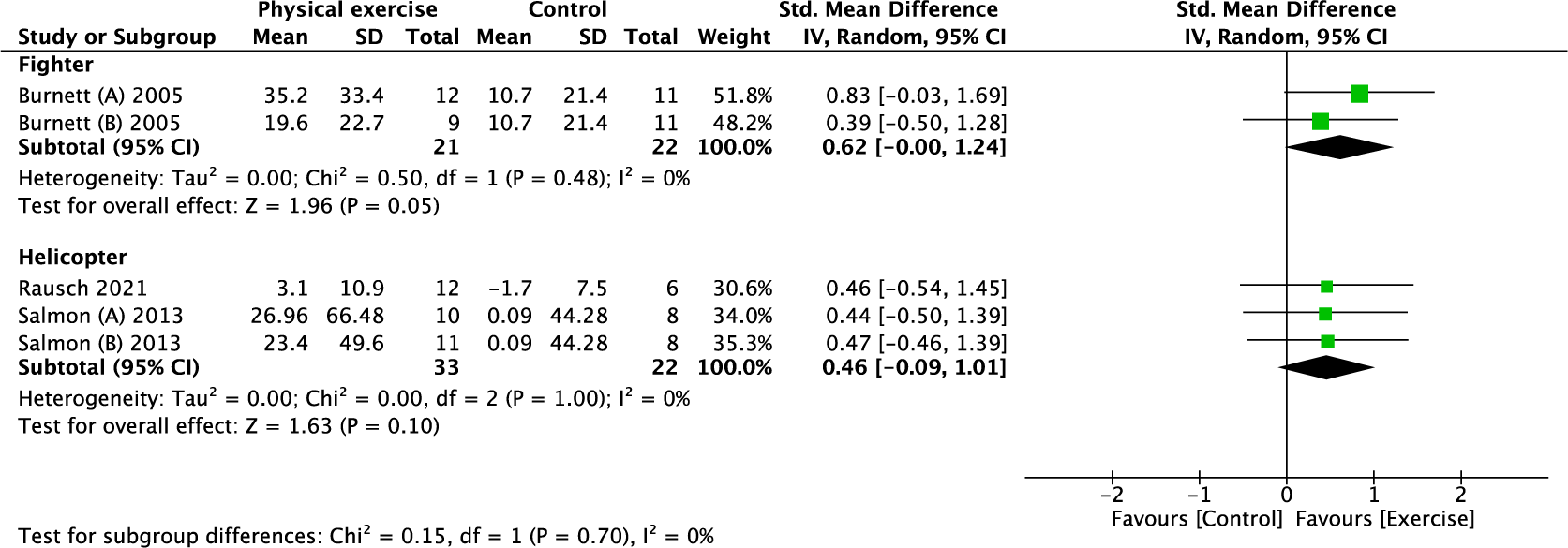


**Supplementary Figure 17.** Subgroup analysis for the MVIC of the neck Ltflx (type of aircraft).


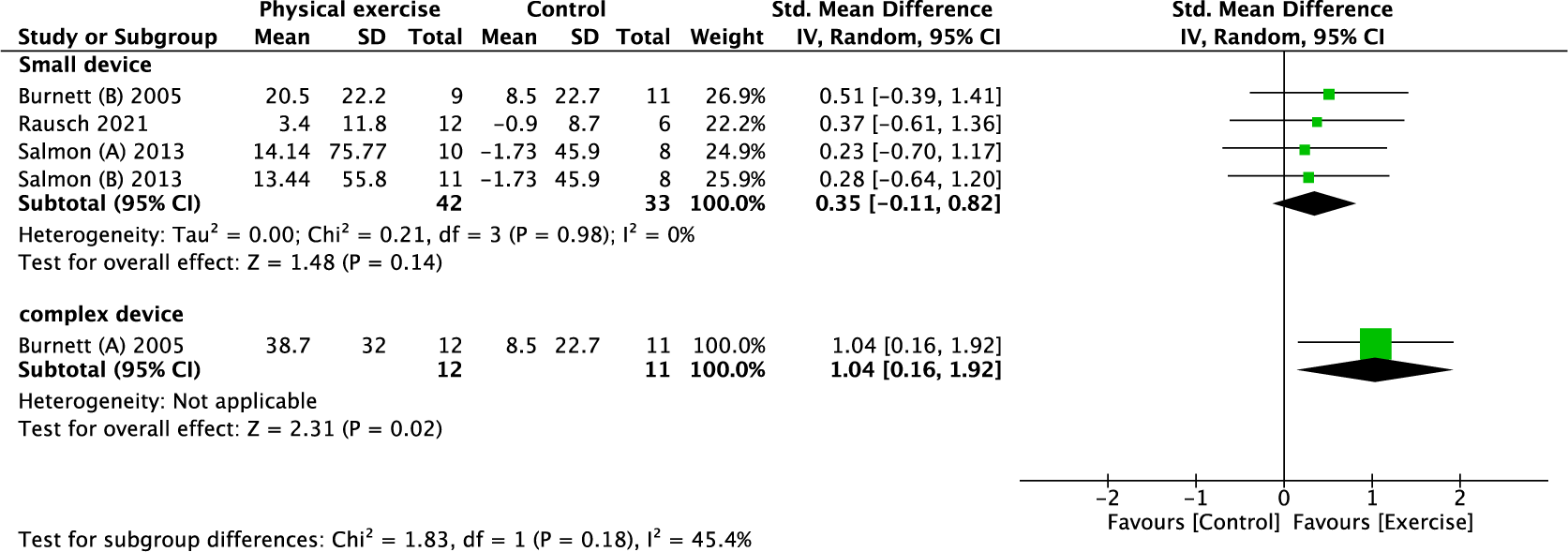


**Supplementary Figure 18.** Subgroup analysis for the MVIC of the neck Ltflx (equipment).


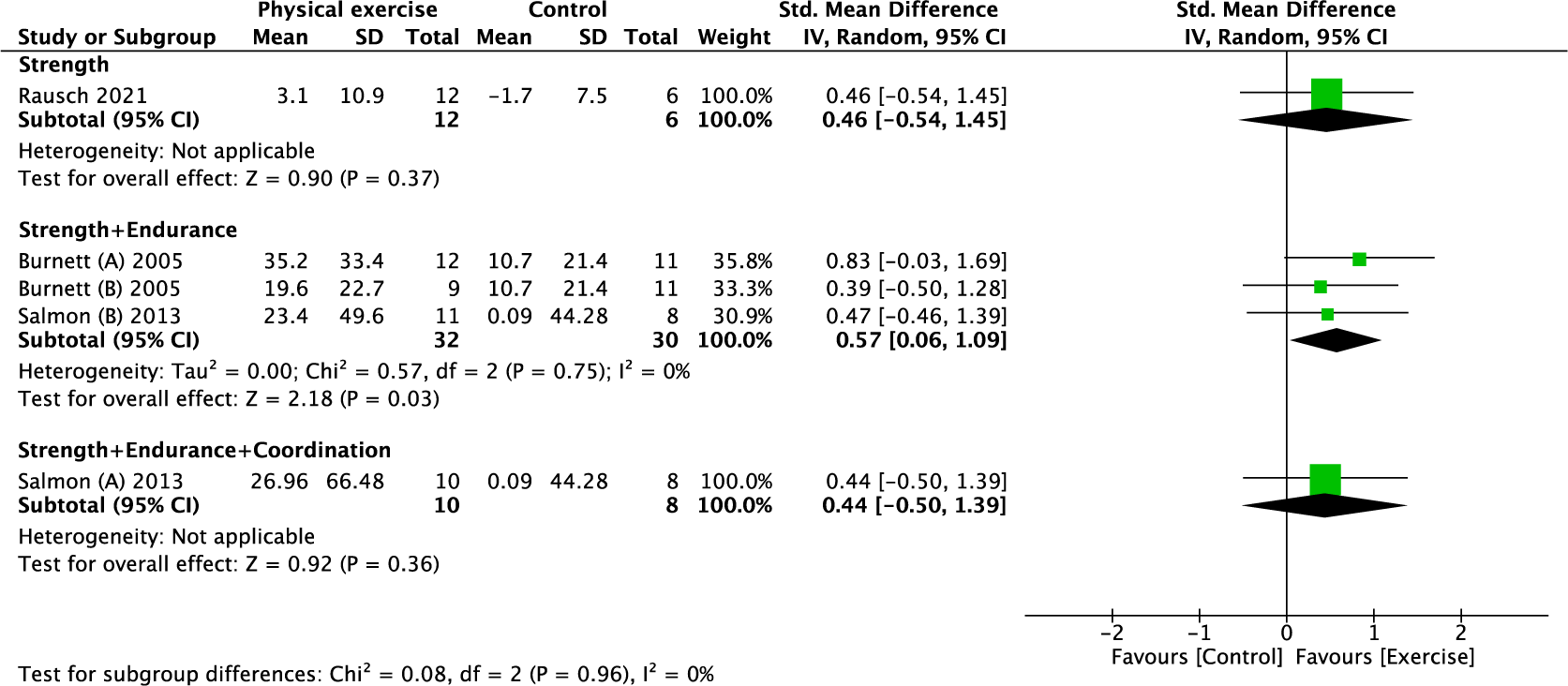


**Supplementary Figure 19.** Subgroup analysis for the MVIC of the neck Ltflx (training protocol).
